# Enhancing Exercise Performance Under Hypoxia: A Network Meta-Analysis and Animal Experimental Validation of Plant Bioactive Compounds

**DOI:** 10.3390/nu18091349

**Published:** 2026-04-24

**Authors:** Huizi Ma, Hongchao Wang, Zhangming Pei, Jianxin Zhao, Hao Zhang, Jing Tian, Wenwei Lu

**Affiliations:** 1 State Key Laboratory of Food Science and Resources, Jiangnan University, Wuxi 214122, China; 6230111073@stu.jiangnan.edu.cn (H.M.); hcwang@jiangnan.edu.cn (H.W.); peizhangming@jiangnan.edu.cn (Z.P.); zhaojianxin@jiangnan.edu.cn (J.Z.); zhanghao61@jiangnan.edu.cn (H.Z.); 2 School of Food Science and Technology, Jiangnan University, Wuxi 214122, China; 3 National Engineering Research Center for Functional Food, Jiangnan University, Wuxi 214122, China; 4 China National Center for Food Safety Risk Assessment, Beijing 100022, China

**Keywords:** hypoxia, exercise performance, plant bioactive compounds, metabolites, functional interventions

## Abstract

Background: Hypoxic environments significantly impair exercise performance, whilst existing interventions are often limited by adverse effects or insufficient efficacy. Objectives and Methods: This study employed network meta-analysis to screen plant bioactive compounds that effectively enhance exercise performance under hypoxic conditions, with subsequent validation of the efficacy and underlying mechanisms of their combined formulations through animal experiments. Results: Results from hypoxic mouse experiments demonstrated that supplementation with the plant bioactive compound combination significantly improved exercise performance, as evidenced by increased weight-loaded swimming time and limb grip strength. Differential metabolite analysis revealed that the intervention altered key metabolic pathways, including the biosynthesis of unsaturated fatty acids and the metabolism of arginine and proline. Supplementation with the plant bioactive compound combination modulated short-chain fatty acid (SCFA) production by gut microbiota, decreased levels of lactic acid (LA), lactate dehydrogenase (LDH), and creatine kinase (CK), maintained blood glucose levels before and after exercise, and increased muscle and hepatic glycogen reserves. These effects collectively improved exercise endurance and performance in mice under hypoxic conditions. Conclusions: The findings provide novel insights into developing functional interventions to enhance exercise performance in hypoxic environments.

## 1. Introduction

Hypoxic environments are natural or artificial settings where atmospheric oxygen levels fall below normal levels. Such environments are commonly encountered in high-altitude regions, certain underground spaces, and specific industrial settings. Plateau environments are characterised by low atmospheric pressure, reduced oxygen levels, low temperatures, and high levels of ultraviolet radiation. At high altitudes, decreased atmospheric pressure and reduced environmental oxygen content lead to hypobaric hypoxia. Physical performance generally declines at high altitudes, severely impacting the daily lives and health of both permanent residents and visitors to plateau regions [1].

In recent years, damage caused by high-altitude hypoxia and its prevention and treatment have attracted widespread attention. Research has demonstrated that the higher the altitude gain, the longer the exercise duration, and the more rapid the ascent, the greater the impact on human exercise performance [2,3]. Both rapid acclimatisation to high altitude and prolonged high-altitude exposure significantly affect human explosive power, endurance, and precision operations. Insufficient oxygen supply at the cellular level impairs an individual’s performance during routine physical activities and challenges such as mountaineering, tourism, and military operations. Prolonged exposure to severe hypoxic environments adversely affects muscle structure [4,5].

Exercise performance refers to the collective manifestation of physical qualities when individuals engage in sport, and is determined by oxygen supply to working muscles and vital organs, including the brain [6]. It is influenced by multiple factors, including reduced energy substrates (glucose and glycogen levels), accumulation of metabolites (such as lactic acid and blood urea nitrogen), alterations in skeletal muscle metabolism, and changes in exercise motivation [7,8]. Excessive exercise causes muscles to enter anaerobic metabolism, leading to lactic acid and peroxide accumulation, inflammation, muscle damage, and neuromuscular fatigue [9,10].

The negative impact of hypoxic environments on exercise performance is largely related to the oxygen delivery capacity of muscles [11,12]. At high altitudes, reduced oxygen levels lead to diminished blood oxygen saturation and weakened aerobic metabolism. This accelerates the transition to anaerobic metabolism, intensifying lactic acid accumulation that damages muscles whilst inducing widespread physiological effects, including oxidative stress damage, significantly diminished oxygen delivery capacity, and reduced exercise endurance [13,14]. Moreover, in the low-oxygen environment of plateaus, altitude sickness is also present, accompanied by issues such as reduced cardiopulmonary function, diminished neurocognitive performance, and decreased maximum oxygen uptake [15,16,17]. Therefore, understanding how to resist hypoxic environments, enhance exercise endurance, improve exercise performance, and alleviate exercise fatigue under plateau hypoxia has become a focal point for researchers.

Current countermeasures addressing the impact of low-oxygen environments on exercise performance primarily focus on pharmaceutical development. Examples include Rhodiola rosea capsules, acetazolamide tablets, and dexamethasone. These agents effectively enhance human tolerance to hypoxia, significantly reducing inflammation and oedema caused by low oxygen levels [18,19,20]. However, these medications often cause a range of adverse effects such as diarrhoea, nausea, and drowsiness, along with the development of resistance. Most dietary supplements targeting hypoxic environments on the market consist of simple ingredients such as glucose, vitamins, and minerals. Whilst glucose provides energy for the body and vitamins and minerals help maintain overall health and enhance resistance, these basic components cannot effectively improve hypoxic tolerance; they only offer mild relief from symptoms such as dizziness and fatigue caused by low oxygen levels. There is a lack of functional products on the market that can effectively enhance human tolerance to hypoxia and resistance to exercise fatigue. Therefore, identifying functional factors with anti-fatigue effects in high-altitude, low-oxygen environments holds significant promise for improving physical work capacity, operational efficiency, and military performance capabilities in such conditions.

Current measures and medications for alleviating fatigue during high-altitude operations have shown limited effectiveness. Whilst certain biochemical drugs can enhance exercise performance, their stimulant-like components may cause bodily harm. Therefore, screening functional compounds from plants that exhibit anti-fatigue and hypoxic tolerance properties with minimal adverse effects represents a more desirable approach [21]. Current research has identified numerous plant active components that can regulate athletic performance under hypoxic conditions by acting on different targets. However, challenges remain in comprehensively screening and evaluating plant active components capable of enhancing athletic performance in hypoxic environments.

In this study, we first employed systematic reviews and network meta-analysis to conduct a meta-screening of plant active components that enhance exercise performance under high-altitude hypoxic conditions. We estimated and ranked their efficacy and formulated combinations of these plant-active components. Second, we established a hypoxic mouse model to investigate the effects of these formulations on exercise performance in hypoxic conditions and to explore their potential mechanisms of action. This study not only reveals the potential of plant active component combinations in enhancing exercise performance under hypoxic conditions but also highlights their practical application value.

## 2. Materials and Methods

### 2.1. Materials

Acetazolamide was obtained from Sanofi Biologics Co., Ltd. (Taizhou, China); Rhodiola was purchased from Yuanye Bio-Technology Co., Ltd. (Shanghai, China); Vitamin D3 was purchased from Sinopharm Xingsha Pharmaceuticals Co., Ltd. (Xiamen, China); All other reagents were purchased from Perfemiker (Shanghai, China).

### 2.2. Search Strategy

We systematically searched PubMed, Web of Science, and additional databases (China National Knowledge Infrastructure, CNKI) for articles published between 2019 and 2024 that examined exercise performance in high-altitude hypoxic environments, focusing on research achievements in which techniques such as hypoxic modelling methods, exercise performance outcome indicators, biochemical assays, and plant trait analysis have become increasingly standardised and significantly improved in comparability over the past five years. We also screened the reference lists of included articles and relevant systematic reviews to identify potentially eligible studies. The complete search strategy is detailed in Section S1.

### 2.3. Eligibility Criteria

We included research or review articles on animal studies (rats/mice) involving hypoxic environments and exercise, in which the intervention/treatment regimen comprised edible bioactive substances administered for at least 1 week. We excluded conference abstracts and the non-English literature, as well as trials lacking a control group or involving non-edible bioactive substance interventions.

### 2.4. Screening Process

We imported the database-retrieved items into EndNote (X9), removed duplicates, and combined them with results from other sources. The screening process consisted of three stages. First, we independently selected articles according to their titles, including any uncertain entries. Second, all articles selected from the first phase underwent abstract review. Third, articles with eligible titles and abstracts were further reviewed in full text according to predetermined inclusion and exclusion criteria.

### 2.5. Data Extraction

For each eligible study, we used predesigned tables to independently extract the following information: study characteristics (year of publication, primary active ingredient, disease type), animals (age, sex, sample size), intervention (name and dose), and outcomes. We measured the changes from baseline in outcomes of pulmonary arterial hypertension (PAH), hypoxia-inducible factor-1α (HIF-1α), erythropoietin (EPO), malondialdehyde (MDA), superoxide dismutase (SOD), glutathione (GSH), lactic acid (LA), glycogen (liver glycogen or muscle glycogen), and lactate dehydrogenase (LDH). We abstracted all relevant data reported in the included trials. We used WebPlot Digitizer version 4.6 to estimate values only when data were presented graphically [22].

### 2.6. Quality Assessment

We used the Systematic Review Center for Laboratory Animal Experimentation (SYRCLE) tool to assess the risk of bias in the included trials, including randomisation method, baseline characteristics, blinding, incomplete outcome data, selective reporting of results, and other biases [23]. If the risk of bias was low in all domains, the overall risk of bias in each trial was considered low (scored as 1); if the risk of bias was high in at least one domain, the overall risk of bias in each trial was considered high (scored as 3). In uncertain circumstances, the risk of bias was considered of some concern (scored as 2). We used funnel plots to scrutinise small-study bias, using estimates of direct evidence, and examined each comparison separately [24].

### 2.7. Data Synthesis and Analysis

We performed network meta-analyses of randomised controlled trials using StataMp 17 (64-bit) and employed the Stata command MVMETA [25] to perform multivariate network meta-analyses within the frequentist framework with a random-effects model [26]. The relative effects were measured as mean differences for continuous outcomes. Random-effects network meta-analyses were estimated using the restricted maximum likelihood method to account for heterogeneity between studies and to calculate pooled estimates and 95% confidence intervals [27]. We used a node-splitting approach to evaluate agreement and inconsistency between direct and indirect estimates within each closed-loop evidence [28,29]. We used Stata to create network diagrams and ranked different intervention protocols based on the surface under the cumulative ranking curve (SUCRA) to evaluate their efficacy in enhancing exercise performance under hypoxic conditions [30].

The present study was not aimed at validating top-ranked single-component compounds, but rather at evaluating the efficacy of combinations selected based on medical compatibility principles following a network meta-analysis. Accordingly, from the substances with the highest efficacy rankings in anti-hypoxia, anti-oxidative stress, and anti-fatigue properties, we selected one or two natural products or nutrients with well-defined activities to form combinations and comprehensively assess their combined effects.

### 2.8. Animal Experiment Design

In total, 36 six-week-old male ICR mice of specific pathogen-free (SPF) grade, with initial body weights ranging from 20 g to 25 g, were purchased from China SPF (Suzhou) Biotechnology Co., Ltd. (Suzhou, China). All animal studies were performed in full compliance with the guidelines approved by the Animal Ethics Committee of Jiangnan University (JN. No20250228i0900601[032]). Mice were housed under controlled conditions (21–26 °C, 40–70% humidity, ≤60 dB noise, 15–20 lux light).

After one week of acclimatisation, the mice were randomly divided into six groups (*n* = 6). The dietary interventions were as follows: (1) Blank (gavage with 0.2 mL of physiological saline); (2) Model (gavage with 0.2 mL of physiological saline); (3) Positive 1 (gavage with 0.2 mL of acetazolamide solution containing 20 mg/kg body weight); (4) Positive 2 (gavage with 0.2 mL of Rhodiola solution containing 100 mg/kg body weight); (5) Intervention 1 (gavage with 0.2 mL of Combination 1 of plant active components, consisting of astragaloside IV, ginsenoside Rg1, puerarin, and vitamin D3); (6) Intervention 2 (gavage with 0.2 mL of Combination 2 of plant active components, consisting of astragalus polysaccharide (APS), gypenoside (GP), moringa leaf extract, and vitamin D3).

Throughout the intervention period, the body weights of the mice were measured weekly. After two weeks of dietary intervention, all groups except the control group underwent two weeks of hypoxic exposure, along with daily 20-minute sessions of weightless swimming training. Throughout this modelling period, the body weights of the mice were recorded every other day. Exercise performance was assessed in the final week, with subsequent collection of faecal samples from each mouse for untargeted metabolomics analysis prior to terminating the experiment. In compliance with animal welfare and ethical guidelines, at the end of the experiment, mice were anaesthetised with isoflurane inhalation anesthesia, and blood was collected from the ophthalmic venous plexus. Following blood collection, the mice were euthanized by cervical dislocation and then dissected for tissue sample collection.

Hypoxia exposure procedure [31]: mice were placed in a hypobaric hypoxic chamber (ProOx-811L-L, Shanghai Tow Intelligent Technology Co., Ltd., Shanghai, China) and raised to a height of 5000 m at a velocity of 500 m/min for 14 days of hypoxia, during which the chamber was opened 2 hours per day for gavage operations, replenishment of food and water, and exercise training. Orbital blood was collected from the mice, and approximately 3–4 drops of whole blood were used to measure the levels of red blood cells (RBC), haemoglobin (HGB), haematocrit (HCT), and mean corpuscular volume (MCV) to evaluate the success of model establishment.

### 2.9. Measurement of Exercise Performance

Following a 30-minute post-gavage rest period, all mice underwent an exhaustive swimming test. They were equipped with a tail load equivalent to 5% of their body weight and swam in water maintained at 25 ± 1 °C. Exhaustion was defined by sluggish hindlimb movement, reduced swimming range, and inability to resurface within 3 seconds after the head sank [32]. Immediately following this weight-loaded swim, blood was collected via the orbital sinus, after which the mice were euthanised.

During the final week of the experiment, limb grip strength was measured using a grip strength metre (Jinan Yiyan Technology Co., Ltd., Jinan, China). Briefly, all four limbs of each mouse were placed horizontally on the grid plate of the device, and the tail was gently pulled backward in a horizontal direction. The test was repeated five times for each animal, and the maximum grip force value was recorded.

### 2.10. Determination of Serum Biochemical Indices

After blood collection, samples were allowed to clot for at least 2 h. Subsequently, they were centrifuged at 3000 rpm for 15 min at 4 °C to separate the serum. The obtained serum was then aliquoted into enzyme-free microcentrifuge tubes, and blood urea (UREA), serum creatinine (SCR), serum calcium (Ca), glucose (Glu), lactate dehydrogenase (LDH), and creatine kinase (CK) levels were measured using a Beckman AU5800 automatic biochemical analyser (Brea, CA, USA).

### 2.11. Measurement of Hypoxia Tolerance, Antioxidant Capacity, and Exercise Fatigue Resistance in Mice

The levels of hypoxia-inducible factor-1α (HIF-1α), erythropoietin (EPO), and vascular endothelial growth factor (VEGF) in serum were detected using enzyme-linked immunosorbent assay (ELISA) kits (mlbio, Shanghai, China) to evaluate hypoxia tolerance.

Malondialdehyde (MDA), superoxide dismutase (SOD), and glutathione (GSH) in serum were determined using specific kits (mlbio, Shanghai, China) following the manufacturer’s instructions. The levels of MDA, SOD, and GSH were assessed to evaluate oxidative stress.

After euthanising the mice, the liver and left gastrocnemius muscle tissues were collected. The collected tissues were washed with phosphate-buffered saline (PBS), promptly frozen in liquid nitrogen, and homogenised at low temperatures. Liver and muscle glycogen and blood lactic acid contents were determined using specific kits (mlbio, Shanghai, China) to evaluate the mice’s resistance to exercise fatigue.

### 2.12. Determination of Short-Chain Fatty Acids (SCFAs) in Mouse Faeces

Approximately 50 mg of faecal sample was homogenised in saturated saline and subjected to liquid–liquid extraction with diethyl ether. The extract was then analysed using an Rtx-Wax capillary column (30 m × 0.25 mm × 0.25 μm) installed on a gas chromatography-mass spectrometry system (GCMS-QP2010 Ultra, Shimadzu, Kyoto, Japan). Nitrogen served as the carrier gas at a constant flow rate of 1.0 mL/min. A 1 μL aliquot of the sample was injected in split mode (split ratio 10:1). The oven temperature was initially held at 100 °C, then ramped at 7.5 °C/min to 140 °C, followed by a second ramp at 60 °C/min to 200 °C, which was maintained for 3 minutes. The injector and ion source temperatures were set at 240 °C and 220 °C, respectively. Quantification was performed using external calibration curves constructed with authentic standards of acetic, propionic, butyric, isobutyric, valeric, and isovaleric acids.

### 2.13. Untargeted Metabolomics Detection and Analysis

For untargeted metabolomic analysis, approximately 20 mg of mouse faecal sample was homogenised in 200 μL of ultrapure water. Subsequently, 800 μL of a pre-chilled (−20 °C) methanol/acetonitrile mixture (1:1, *v*/*v*) was added, followed by vortexing for 30 s. The mixture was then subjected to ultrasound-assisted extraction in an ice bath (4 °C) for 10 min, a process that was repeated twice. To enhance protein precipitation efficiency, the sample was incubated at −20 °C for 1 hour. Following incubation, it was centrifuged at 15,000 rpm for 15 min at 4 °C. The resulting supernatant was collected and dried using a vacuum centrifugal concentrator (Thermo Fisher Scientific, Waltham, MA, USA). The dried residue was reconstituted in 100 μL of an acetonitrile/water solution (1:1, *v*/*v*) by vortexing for 30 s. After a final centrifugation step under the same conditions (15,000 rpm, 15 min, 4 °C), the clarified supernatant was filtered through a 0.22-μm membrane and transferred into an autosampler vial for instrumental analysis.

A 50 μL serum sample was mixed with 200 μL of pre-chilled methanol/acetonitrile (1:1, *v*/*v*) stored at −20 °C to precipitate proteins, and vortexed for 30 s to ensure thorough mixing of the sample with the extraction solvent. Subsequently, the mixture was subjected to ice-bath (4 °C) sonication for 10 min. The sample was incubated at −20 °C for 1 h to improve protein precipitation efficiency, followed by high-speed centrifugation at 15,000 rpm for 15 min at 4 °C. The resulting supernatant was transferred to a sample vial for subsequent instrumental analysis. A Vanquish (Thermo Fisher Scientific) ultra-high-performance liquid chromatography system with a Waters ACQUITY UPLC BEH Amide column (Waters Corporation, Milford, CT, USA) was used for chromatographic separation of the target compounds. Primary and secondary mass spectrometry data acquisition and analysis were performed using an Orbitrap Exploris 120 Mass Spectrometer (Thermo Fisher Scientific, Milford, CT, USA) under the control of Xcalibur software 4.1.

### 2.14. Statistical Analysis

Statistical analysis and data visualisation were conducted using GraphPad Prism (v. 8.4.3), and data are expressed as mean ± standard deviation (SD). When the assumptions of normal distribution and homogeneity of variances were met, differences in means between groups were analysed by one-way analysis of variance (ANOVA) with Duncan’s multiple range test. If the assumptions were violated, nonparametric analysis was employed instead. Statistically significant differences between experimental groups were evaluated using GraphPad Prism software. We used # and * for analysis of variance, comparing the model group to the blank group (significant differences denoted by #) and the intervention group to the model group (significant differences denoted by *), whilst no markings denote no significance. A *p*-value < 0.05 was considered statistically significant. * *p* < 0.05, ** *p* < 0.01, *** *p* < 0.001, and **** *p* < 0.0001.

## 3. Results

### 3.1. Search and Inclusion Results

**Figure 1:**
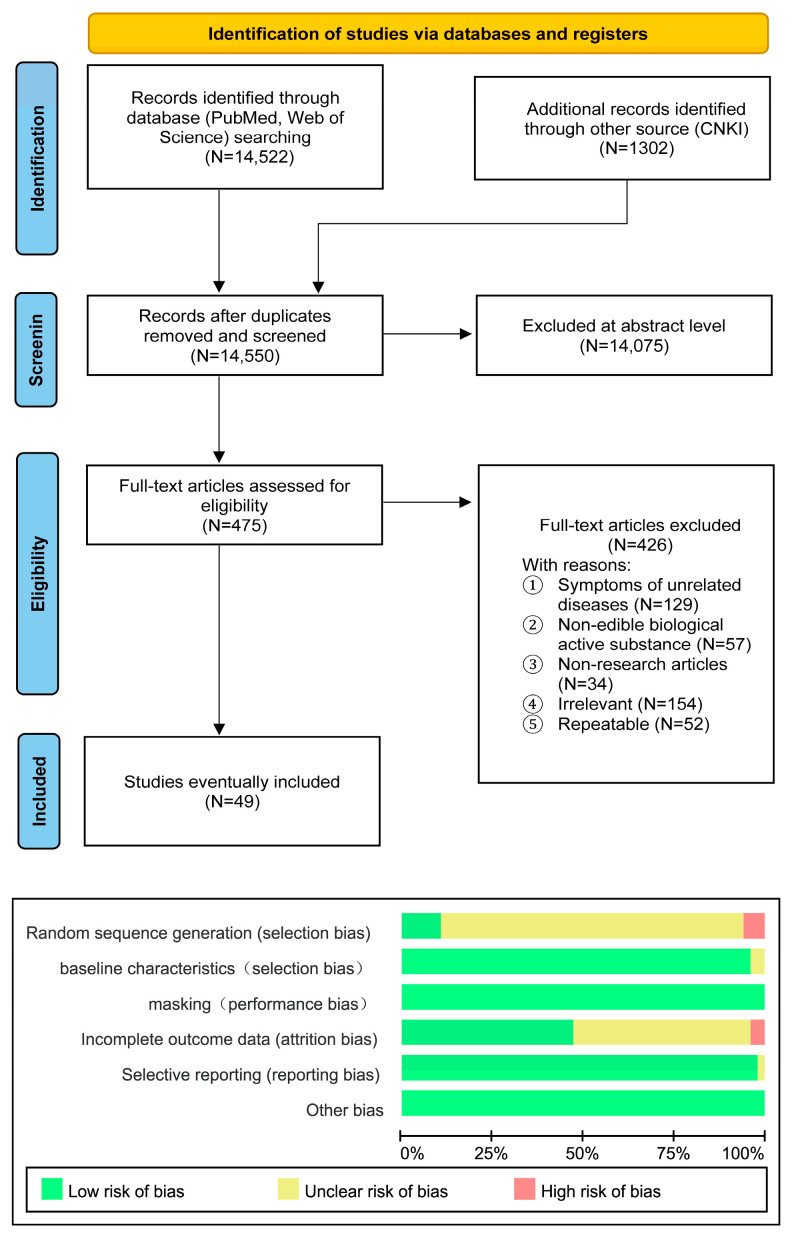
Flow diagram of preferred reporting items identified, included, and excluded for systematic reviews and meta-analyses (PRISMA), and a risk of bias graph for the eventually included studies. The overall risk of bias is presented as a percentage for each risk of bias item across all included studies. Green = Low risk, Red = High risk, Yellow = Some concerns.

Our electronic search identified 14,550 potentially relevant publications (Figure 1). After excluding duplicates and screening titles and abstracts, we retrieved 475 publications for full-text review. Among these, 129 articles pertained to disease symptoms unrelated to the research, 57 addressed non-edible biologically active substances, 34 were non-research articles, 154 contained irrelevant content, and 52 were duplicates. Following exclusion, 49 articles were ultimately included. Data extraction and risk of bias assessment were conducted on the included articles (Figure 1). In 49 trials, 3 studies (6%) had a high risk of random sequence generation bias, 47 studies (96%) had a low risk of baseline characteristics bias, 49 studies (100%) had a low risk of masking bias, 23 studies (47%) had a low risk of incomplete outcome data bias, 48 studies (98%) had a low risk of selective reporting bias, and 49 studies (100%) had a low risk for other bias. Overall, 5 studies (10%) had a high risk of bias.

### 3.2. Evaluation of Inconsistency

For our consistency evaluation (i.e., alignment of direct and indirect evidence), results suggested inconsistency in several comparisons (Table S1); however, no strong statistical evidence of global inconsistency was reported for most outcomes. Additionally, inconsistency testing between direct and indirect intervention comparisons was performed for each experimental result. The findings (Section S2) revealed inconsistencies in a small number of intervention comparisons, but the majority showed no significant inconsistencies. Furthermore, we found no evidence of asymmetry in the funnel plots (Section S3).

### 3.3. Network Meta-Analysis Results of Hypoxic Tolerance Indicators Under Different Interventions

**Figure 2:**
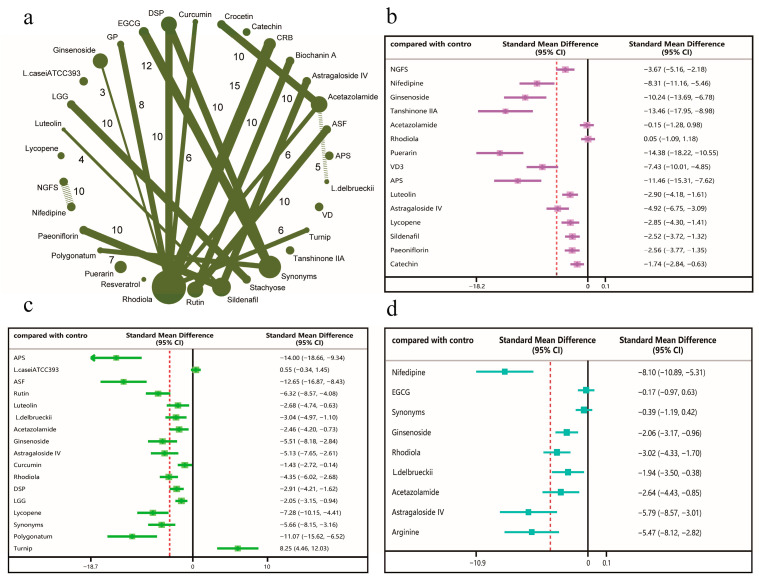
Network meta-analysis results of hypoxic tolerance indicators under different interventions (The red dashed line in the figure represents the overall mean of the results obtained from the different interventions). (**a**) Network map: each dot represents an intervention, with dot size indicating sample size. Lines connecting dots denote pairwise comparisons conducted in studies, with line thickness reflecting the number of comparative studies. (**b**) Forest plot of network effect sizes for PAH. (**c**) Forest plot of network effect sizes for HIF-1α. (**d**) Forest plot of network effect sizes for EPO.

We categorised indicators related to exercise performance in high-altitude hypoxic environments: HIF-1α, EPO, and PAH were classified as hypoxic tolerance indicators; MDA, SOD, and GSH were classified as antioxidant stress indicators; and LA, glycogen, and LDH were classified as fatigue resistance indicators. Regarding hypoxic tolerance, the network meta-analysis comprised 30 studies (Figure 2a). For the PAH random forest results (Figure 2b), most active compounds reduced PAH levels, with significant reductions exhibited particularly by puerarin and tanshinone IIA. For the HIF-1α random forest results (Figure 2c), Astragalus polysaccharide (APS), Aster souliei Franch (ASF), and polygonatum significantly reduced HIF-1α levels, whilst turnip significantly increased HIF-1α levels. For the EPO random forest results (Figure 2d), nifedipine, astragaloside IV, and arginine were found to reduce EPO levels, whilst EGCG and its synonyms had little effect on EPO levels.

### 3.4. Network Meta-Analysis Results of Antioxidant Stress Resistance Indicators Under Different Interventions

**Figure 3:**
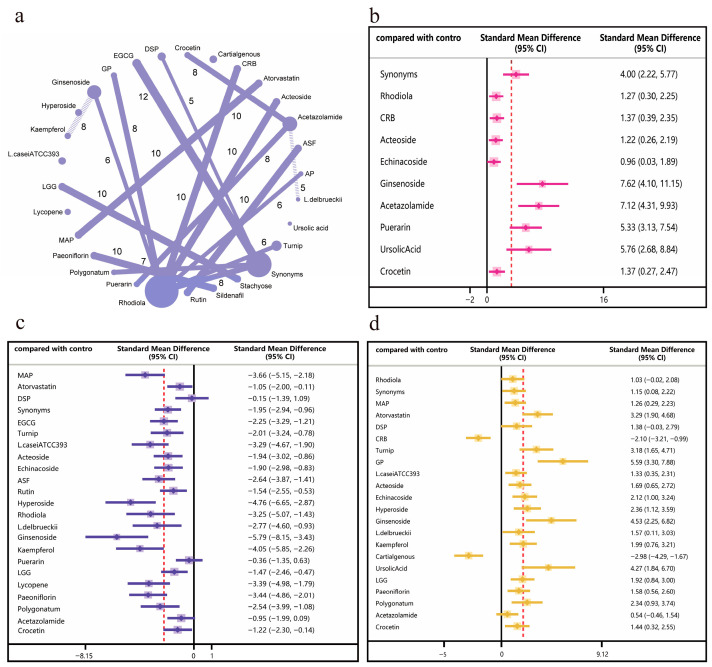
Network meta-analysis results of antioxidant stress resistance indicators under different interventions (The red dashed line in the figure represents the overall mean of the results obtained from the different interventions). (**a**) Network map: each dot represents an intervention, with dot size indicating sample size. Lines connecting dots denote pairwise comparisons conducted in studies, with line thickness reflecting the number of comparative studies. (**b**) Forest plot of network effect sizes for GSH. (**c**) Forest plot of network effect sizes for MDA. (**d**) Forest plot of network effect sizes for SOD.

Regarding antioxidant stress, the network meta-analysis comprised 28 studies (Figure 3a). For the GSH random forest results (Figure 3b), ginsenosides and acetazolamide significantly elevated GSH levels. For the MDA random forest results (Figure 3c), 11 interventions effectively reduced MDA levels, with 7 interventions showing modest decreases. Among these, ginsenoside and hyperoside were particularly effective in lowering MDA levels. For the SOD random forest results (Figure 3d), most interventions increased SOD levels, with only two interventions reducing SOD levels. GP and ginsenoside demonstrated superior efficacy in enhancing SOD levels.

### 3.5. Network Meta-Analysis Results of Indicators of Resistance to Exercise Fatigue Under Different Interventions

**Figure 4:**
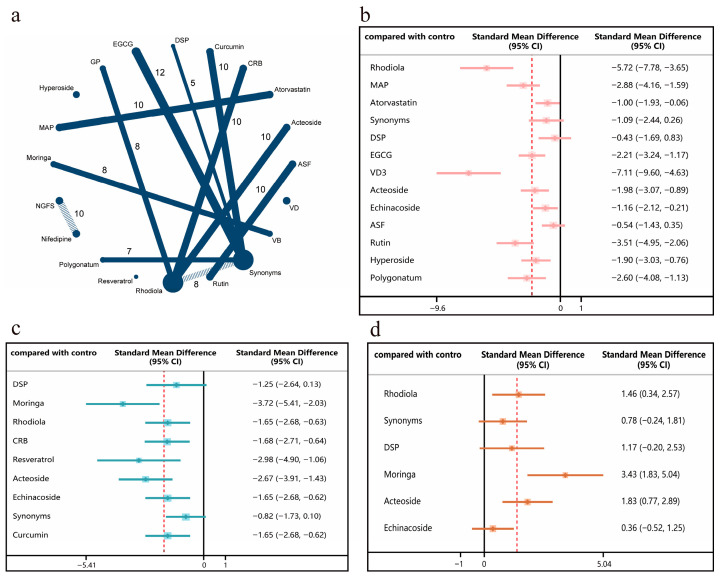
Network meta-analysis results of indicators of resistance to exercise fatigue under different interventions (The red dashed line in the figure represents the overall mean of the results obtained from the different interventions). (**a**) Network map: each dot represents an intervention, with dot size indicating sample size. Lines connecting dots denote pairwise comparisons conducted in studies, with line thickness reflecting the number of comparative studies. (**b**) Forest plot of network effect sizes for LDH. (**c**) Forest plot of network effect sizes for LA. (**d**) Forest plot of network effect sizes for Glycogen.

Regarding resistance to exercise fatigue, the network meta-analysis comprised 18 studies (Figure 4a). For the LDH random forest results (Figure 4b), most interventions increased LDH levels, whilst vitamin D3 significantly reduced LDH levels. For the LA random forest results (Figure 4c), moringa (moringa leaf extract) and resveratrol significantly reduced LA levels. For the glycogen random forest results (Figure 4d), moringa (moringa leaf extract) demonstrated an effect of increasing glycogen levels.

### 3.6. SUCRA and Cumulative Probability Plots Based on Active Component Efficacy Ranking

**Figure 5:**
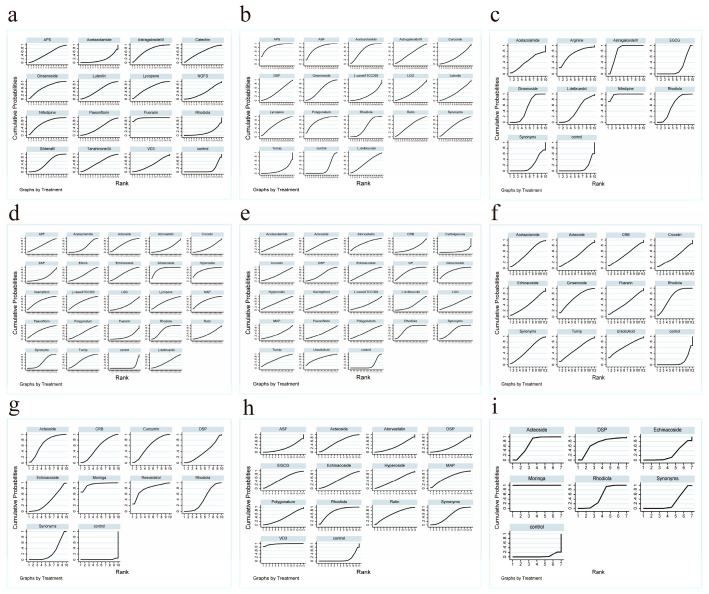
SUCRA and cumulative probability plots. (**a**) Cumulative ranking curve plots of different active components for PAH in the range network. (**b**) Cumulative ranking curve plots of different active components for HIF-1α in the range network. (**c**) Cumulative ranking curve plots of different active components for EPO in the range network. (**d**) Cumulative ranking curve plots of different active components for MDA in the range network. (**e**) Cumulative ranking curve plots of different active components for SOD in the range network. (**f**) Cumulative ranking curve plots of different active components for GSH in the range network. (**g**) Cumulative ranking curve plots of different active components for LA in the range network. (**h**) Cumulative ranking curve plots of different active components for LDH in the range network. (**i**) Cumulative ranking curve plots of different active components for Glycogen in the range network. A higher surface under the curve indicates a higher probability of association with these indicators.

To more intuitively compare the effects of different plant bioactive compound-based interventions on improving exercise performance indicators under hypoxic conditions, SUCRA and cumulative probability plots were used to evaluate and rank the efficacy of these compounds (Figure 5). The SUCRA score ranges between 0% and 100%. A higher score corresponds to greater intervention efficacy and a more favourable ranking among the compared bioactive compounds. The results showed that puerarin was the most effective in alleviating pulmonary arterial hypertension and reducing PAH levels; Astragalus polysaccharides (APS) were optimal for maintaining HIF-1α levels; astragaloside IV performed best in preventing excessive elevation of EPO; ginsenoside was most effective in lowering MDA levels and increasing GSH levels; gypenosides (GP) showed the highest efficacy in elevating SOD levels; moringa leaf extract was superior in reducing lactate accumulation and increasing glycogen storage; whilst vitamin D3 (VD3) played a beneficial role in improving muscle function, stabilising LDH levels, and reducing muscle damage.

More detailed comparisons of these active components were provided in SUCRA data (Section S4) and a table (Section S5).

### 3.7. Effects of Plant Active Component Combinations on Blood Cells in Hypoxic Mice

**Figure 6:**
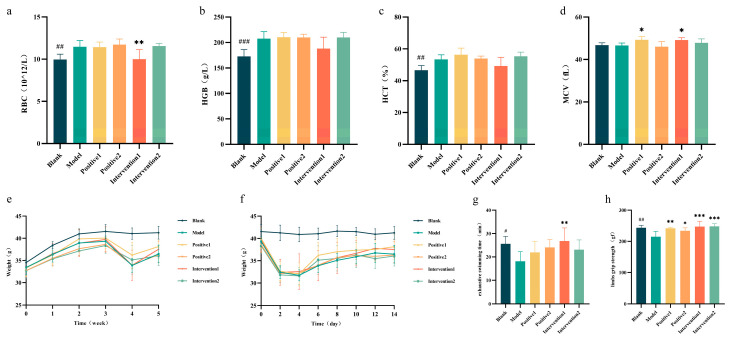
Effects of plant active component combinations on blood cells and exercise performance in hypoxic mice. (**a**) Red blood cell (RBC) count. (**b**) Haemoglobin (HGB) count. (**c**) Haematocrit (HCT). (**d**) Mean corpuscular volume (MCV). (**e**) Weight: weekly measurement. (**f**) Weight: daily measurement. (**g**) Exhaustive swimming time. (**h**) Limb grip strength. A *p*-value < 0.05 was considered statistically significant. * *p* < 0.05, ** *p* < 0.01, and *** *p* < 0.001. # *p* < 0.05, ## *p* < 0.01, and ### *p* < 0.001.

A hypoxic mouse model was established by housing mice in a low-pressure chamber mimicking conditions at 5000 m altitude. Analysis of whole-blood data (Figure 6a–d) showed that the model group had significantly elevated red blood cell (RBC) count, haemoglobin (HGB), and haematocrit (HCT) compared with the blank group, validating successful model induction. Notably, Intervention 1 reduced these parameters back towards blank levels.

### 3.8. Effects of Plant Active Component Combinations on Exercise Performance in Hypoxic Mice

Compared with the blank group, mice in the low-pressure oxygen chamber exhibited reduced locomotor frequency, primarily manifesting as lethargy and decreased mental alertness, along with reduced food and water intake. Analysis of body weight changes (Figure 6e) showed no significant differences among groups during the first three weeks. During the fourth week, when hypoxic modelling was initiated, all groups except the blank group showed a significant decrease in body weight. By the fifth week, body weight had begun to recover across groups, with Positive 1 and Intervention 1 demonstrating pronounced restorative effects on weight recovery. Examining the precise modelling timeline (Figure 6f), a marked decline in body weight was observed across all groups two days after modelling began. Starting on the third day, mice in Intervention 1 began to show weight recovery, followed by a steady increasing trend. The remaining groups started to regain weight after the fourth day.

To further investigate the effects of plant bioactive compounds on exercise performance in hypoxic mice, the animals underwent 4-week gavage administration of plant active component combinations. Following 2 weeks of swimming training, exhaustive swimming time (Figure 6g) and limb grip strength (Figure 6h) were assessed. Compared with the blank group (25.61 ± 3.198 min, 243.6 ± 7.905 gf), the model group (18.14 ± 4.100 min, 215.0 ± 17.17 gf) demonstrated significantly reduced exhaustive swimming time and limb grip strength. Conversely, Intervention 1 (26.83 ± 5.591 min, 247.3 ± 17.43 gf) significantly enhanced both exhaustive swimming time (*p* < 0.01) and limb grip strength (*p* < 0.001) compared with the model group. Similarly, Positive 1, Positive 2, and Intervention 2 also improved limb grip strength in hypoxic mice.

### 3.9. Effects of Plant Active Component Combinations on Serum Biochemical Parameters in Hypoxic Mice

**Figure 7:**
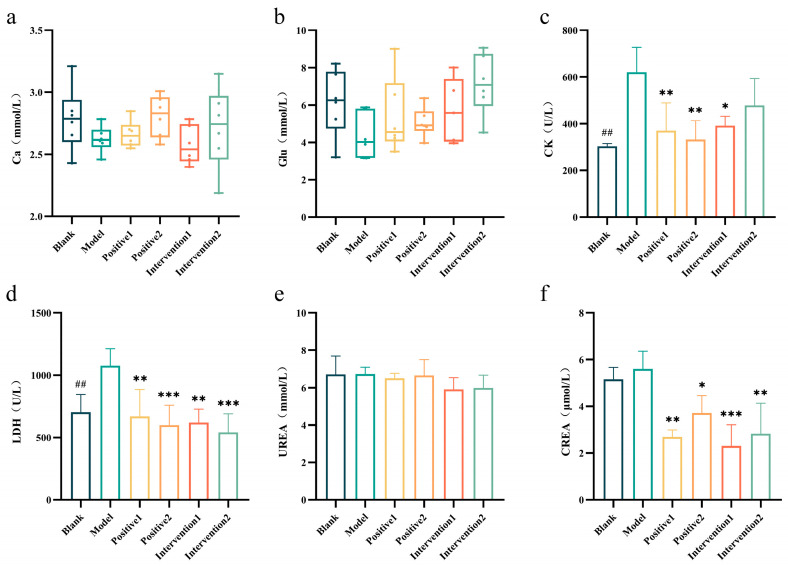
Effects of plant active component combinations on serum biochemical parameters in hypoxic mice. (**a**) Ca level. (**b**) Glucose (Glu) level. (**c**) Serum creatine kinase (CK) level. (**d**) Serum lactate dehydrogenase (LDH) level. (**e**) Blood urea (UREA) level. (**f**) Blood creatinine (CREA) level. A *p*-value < 0.05 was considered statistically significant. * *p* < 0.05, ** *p* < 0.01, and *** *p* < 0.001. ## *p* < 0.01.

To evaluate exercise-induced fatigue, relevant serum biochemical markers were assessed. The experimental results (Figure 7a–f) demonstrated that following exhaustive swimming, the model group exhibited decreased levels of blood calcium (Ca) and blood glucose (Glu) compared with the blank group, suggesting that exercise under hypobaric hypoxia may induce hypoglycaemia. Additionally, the model group showed significantly elevated serum fatigue-related indicators, including lactate dehydrogenase (LDH) and creatine kinase (CK) levels, compared with the blank group after exhaustive swimming. Intervention 1 significantly reduced both LDH (*p* < 0.01) and CK (*p* < 0.05) levels in mouse serum compared with the model group. Notably, all groups except the blank group exhibited significantly decreased blood creatinine levels compared with the model group.

### 3.10. Effects of Plant Active Component Combinations on Hypoxia Tolerance, Antioxidant Stress, and Exercise Fatigue Resistance in Mice

**Figure 8:**
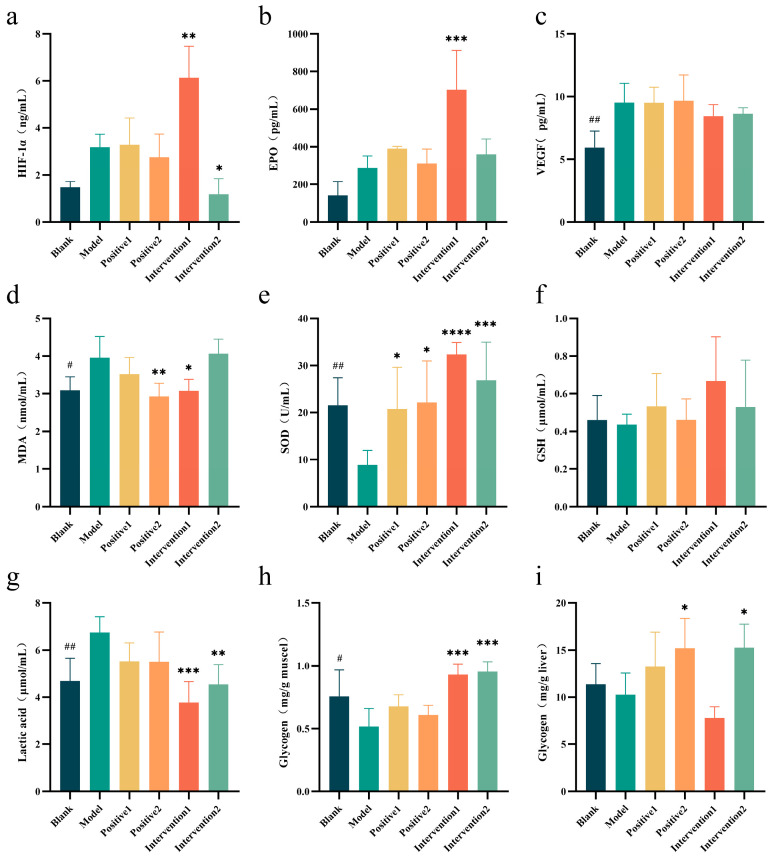
Effects of plant active component combinations on hypoxia tolerance, antioxidant stress, and exercise fatigue resistance in mice. (**a**) Hypoxia-inducible factor-1α (HIF-1α) level. (**b**) Erythropoietin (EPO) level. (**c**) Vascular endothelial growth factor (VEGF) level. (**d**) Malondialdehyde (MDA) level. (**e**) Superoxide dismutase (SOD) level. (**f**) Glutathione (GSH) level. (**g**) Lactic acid level. (**h**) Muscle glycogen level. (**i**) Liver glycogen level. A *p*-value < 0.05 was considered statistically significant. * *p* < 0.05, ** *p* < 0.01, *** *p* < 0.001, and **** *p* < 0.0001. # *p* < 0.05 and ## *p* < 0.01.

To explore the comprehensive effects of plant bioactive compound combinations on exercise performance under hypoxic conditions, assessments were conducted across three domains: hypoxia tolerance, antioxidant stress capacity, and exercise fatigue resistance. Regarding hypoxia tolerance (Figure 8a–i), Intervention 1 significantly increased protein levels of both hypoxia-inducible factor-1α (HIF-1α) (*p* < 0.01) and erythropoietin (EPO) (*p* < 0.001) compared with the model group, whilst Intervention 2 showed no detectable HIF-1α protein expression. Concerning antioxidant stress capacity, the model group exhibited significantly increased malondialdehyde (MDA) content and decreased superoxide dismutase (SOD) levels compared with the blank group. In contrast, Intervention 1 significantly reduced MDA production whilst markedly increasing SOD content compared with the model group. Positive 1, Positive 2, and Intervention 2 also significantly elevated SOD levels. Regarding exercise fatigue resistance, the model group demonstrated significantly reduced muscle glycogen and liver glycogen reserves alongside marked lactate accumulation compared with the blank group. Intervention 1 (*p* < 0.001) and Intervention 2 (*p* < 0.01) significantly decreased lactate accumulation and restored muscle glycogen content (*p* < 0.001) compared with the model group. Furthermore, Positive 2 and Intervention 2 effectively restored liver glycogen levels.

### 3.11. Effects of Plant Active Component Combinations on Short-Chain Fatty Acids in Hypoxic Mice

**Figure 9:**
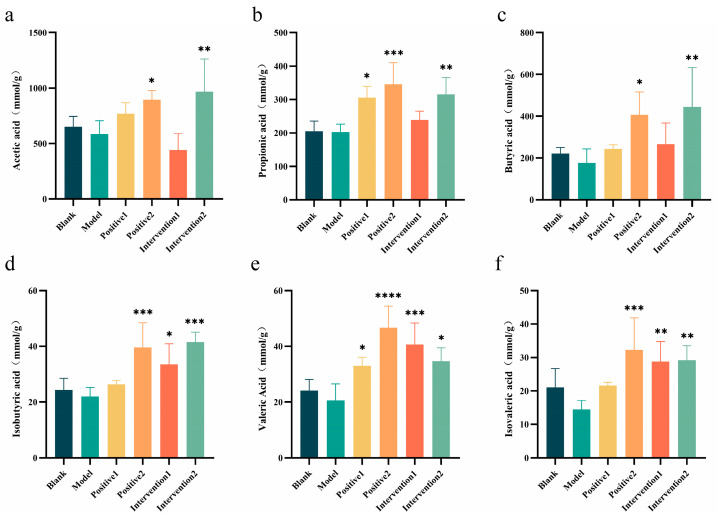
Effects of plant active component combinations on short-chain fatty acids (SCFAs) in the gut metabolites of hypoxic mice. (**a**) Acetic acid level. (**b**) Propionic acid level. (**c**) Butyric acid level. (**d**) Isobutyric acid level. (**e**) Valeric acid level. (**f**) Isovaleric acid level. A *p*-value < 0.05 was considered statistically significant. * *p* < 0.05, ** *p* < 0.01, *** *p* < 0.001, and **** *p* < 0.0001.

To further elucidate the mechanism by which plant bioactive compound combinations improve exercise performance in hypoxic mice, short-chain fatty acid (SCFA) levels in mouse faeces were measured. The SCFA results (Figure 9a–f) revealed that the model group exhibited reduced contents of acetate, propionate, butyrate, isobutyrate, valerate, and isovalerate compared with the blank group. This decrease in intestinal SCFA levels may be attributed to the combined effects of hypoxia and high-intensity exercise. However, administration of positive controls and plant bioactive compound blends ameliorated this decline in SCFA content. Specifically, Positive 2 and Intervention 2 significantly increased levels of all measured SCFAs, whilst Intervention 1 notably elevated isobutyrate, valerate, and isovalerate contents.

### 3.12. Effects of Plant Active Component Combinations on Faecal and Serum Untargeted Metabolomes in Hypoxic Mice

**Figure 10:**
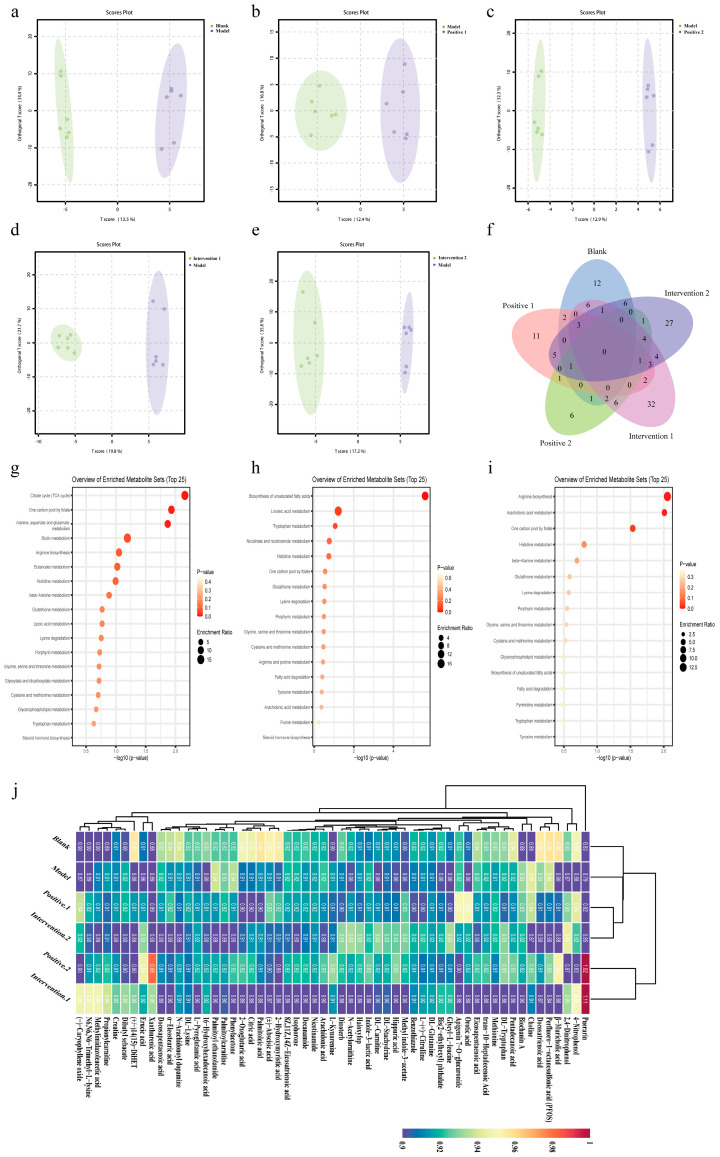
Effects of combinations of plant active components on the faecal untargeted metabolome of mice exercising under hypoxic conditions. (**a**–**e**) Scatterplot of OPLS-DA model score. (**f**) Common significantly different metabolites amongst metabolic sets revealed by the Venn diagram. (**g**–**i**) KEGG functional pathway analysis of differential metabolites. (**j**) Heatmap of hierarchical clustering analysis for significantly different metabolites across groups.

Given that plant bioactive compound combinations can increase gut microbiota-derived SCFAs, untargeted metabolomics was performed on mouse faeces to further characterise broader alterations in intestinal metabolites. The extent of between-group differences in metabolites was analysed using orthogonal projections to latent structures-discriminant analysis (OPLS-DA). Significant differences were observed between the blank and model groups, between the model and positive groups, and between the model and intervention groups (Figure 10a–e). The Venn diagram (Figure 10f) reveals the common metabolites significantly altered in all groups compared to the model group, whilst the heatmap (Figure 10j) depicts the differential abundance patterns of these metabolites across groups.

Analysis based on the Kyoto Encyclopaedia of Genes and Genomes (KEGG) database indicated that significantly altered metabolites between the blank and model groups primarily involved reductions in citric acid, oxoglutaric acid, methionine, and butyric acid, alongside increased choline in the faecal samples of the model group—a finding consistent with the targeted SCFA results. KEGG pathway enrichment analysis of the differential metabolites identified the “Citrate cycle (TCA cycle)”, “One carbon pool by folate”, and “Alanine, aspartate and glutamate metabolism” as the key pathways most affected in the model group (Figure 10g). Compared with the model group, Intervention 1 showed a distinct metabolic profile characterised by reduced faecal levels of oleic acid, arachidonic acid, and tryptophan, alongside elevated levels of isobutyrate and valerate. The key enriched pathways were “Biosynthesis of unsaturated fatty acids” and “Tryptophan metabolism” (Figure 10h). Significantly different metabolites between the model group and Intervention 2 primarily involved increased faecal contents of citrulline, N2-acetylornithine, acetate, propionate, and butyrate in Intervention 2. The key metabolic pathways predominantly enriched in Intervention 2 were “Arginine biosynthesis” and “Arachidonic acid metabolism” (Figure 10i).

**Figure 11:**
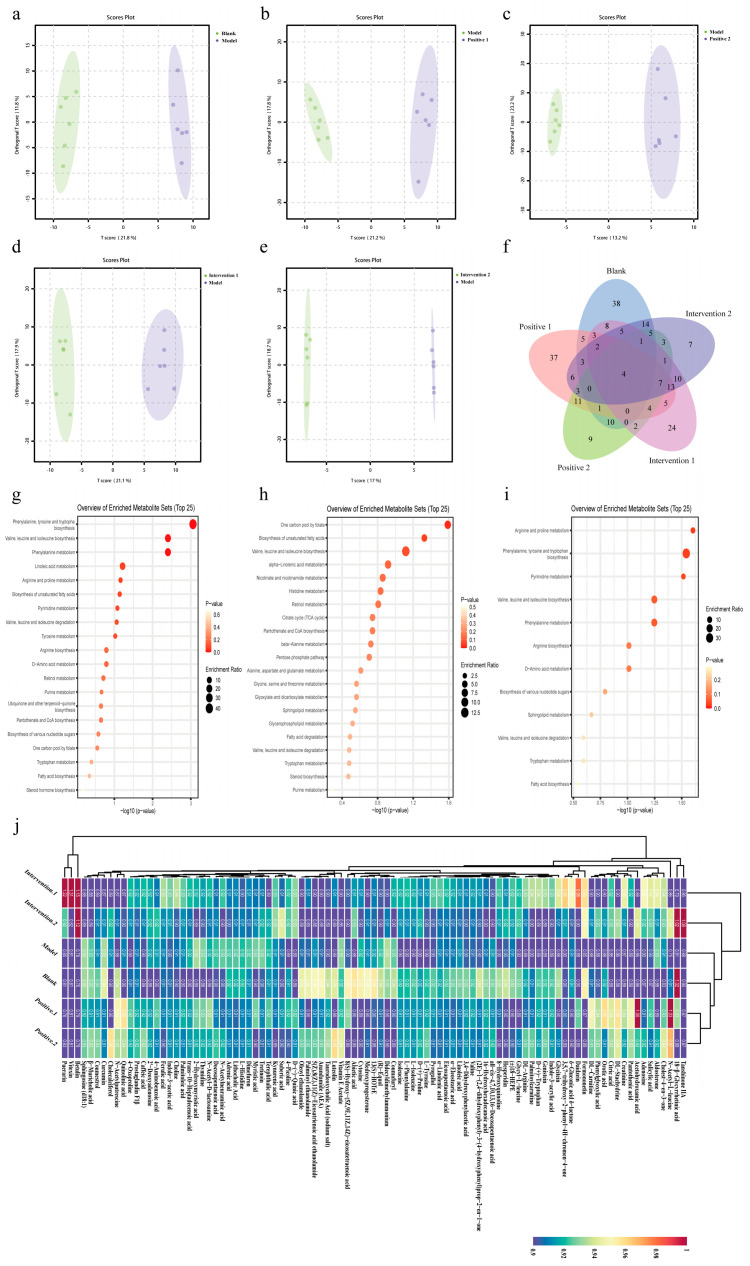
Effects of combinations of plant active components on serum untargeted metabolome of mice exercising under hypoxic conditions. (**a**–**e**) Scatterplot of OPLS-DA model score. (**f**) Common significantly different metabolites amongst metabolic sets revealed by the Venn diagram. (**g**–**i**) KEGG functional pathway analysis of differential metabolites. (**j**) Heatmap of hierarchical clustering analysis for significantly different metabolites across groups.

The untargeted faecal metabolomics data demonstrated that intervention with plant bioactive compound combinations modulated intestinal metabolism, prompting further untargeted metabolomic profiling of mouse serum. Significant differences were observed between the blank and model groups, between the model and positive groups, and between the model and intervention groups (Figure 11a–e). The model group exhibited reprogramming of amino acid metabolism relative to the blank group, characterised by perturbations in the “Phenylalanine, tyrosine and tryptophan biosynthesis” and “Valine, leucine and isoleucine biosynthesis” pathways. KEGG-based analysis specifically identified decreased serum concentrations of phenylalanine, L-tyrosine, isoleucine, and L-valine (Figure 11g). Compared to the model group, the key metabolic pathways altered in Intervention 1 were “One carbon pool by folate” and “Biosynthesis of unsaturated fatty acids”. Based on the KEGG database, analysis of differential metabolites revealed that serum levels of adenosine, choline, eicosapentaenoic acid, linolenic acid, and isoleucine were increased in Intervention 1 (Figure 11h). The metabolic profile of Intervention 2 was characterised by modulation of the “Arginine and proline metabolism” and “Phenylalanine, tyrosine and tryptophan biosynthesis” pathways. KEGG-based analysis indicated this involved elevated serum levels of L-arginine, N-acetylputrescine, phenylalanine, and orotic acid, with a concomitant decrease in thymidine compared to the model group (Figure 11i).

## 4. Discussion

This network meta-analysis comprehensively evaluates and compares the functional efficacy of various plant-derived active ingredients in enhancing exercise performance under hypoxic conditions from three key perspectives: hypoxia tolerance, anti-oxidative stress, and anti-exercise fatigue. It also incorporates positive control drugs (such as acetazolamide and Rhodiola) used in the included studies. Through 49 eligible randomised controlled trials (involving a total of 1,455 laboratory animals), the effects of various plant-derived active ingredients on PAH, HIF-1α, EPO, MDA, SOD, GSH, LA, glycogen, and LDH were evaluated. Nevertheless, despite the use of the SYRCLE tool for risk-of-bias assessment, heterogeneity persists across the 49 included studies due to varied animal models, disparate doses, and mixed outcomes. With this caveat in mind, we further summarise and compare the efficacy rankings of the included active ingredients. Based on the aggregated data, most active ingredients demonstrate certain effects compared to the model group. In terms of enhancing hypoxia tolerance, puerarin, astragaloside IV, and Astragalus polysaccharide (APS) exhibit the most significant efficacy. Regarding enhancing antioxidant stress capacity, ginsenosides and gypenosides (GP) demonstrate the most prominent effects. For alleviating exercise-induced fatigue and improving exercise performance, Moringa leaf extract and vitamin D3 (VD3) are highly effective. Therefore, we combined the most effective plant-derived active ingredients to formulate two composite formulations (Formula 1: astragaloside IV, ginsenoside Rg1, puerarin, VD3; Formula 2: APS, GP, Moringa leaf extract, VD3), and conducted exploratory research and validation on their efficacy. Physiologically, this combination strategy was designed to target multiple pathological processes underlying hypoxic injury, including insufficient oxygen utilisation, oxidative stress damage, energy metabolism disorders, and fatigue-related metabolic disturbances. Each component was selected to complement the others by modulating hypoxia-responsive signalling, scavenging reactive oxygen species, maintaining mitochondrial function, and improving physical endurance under high-altitude stress. On this basis, we performed exploratory investigations and experimental validation to evaluate their combined efficacy.

The high-altitude hypoxic environment often triggers high-altitude sickness, manifesting as dizziness, headache, loss of appetite, lethargy, and susceptibility to fatigue [33], whilst concurrently leading to diminished physical function, increased fatigue, and reduced exercise performance [34]. The 2-week modelling period in this study was designed to capture short-term functional responses under simulated hypoxic conditions. In this study, we observed that intervention with plant-derived active ingredient combinations accelerated weight recovery in mice. Specifically, the formulation comprising astragaloside IV, ginsenoside Rg1, puerarin, and VD3 demonstrated a significant effect on promoting weight recovery in hypoxic mice. Exhaustion swimming time serves as an indicator of anti-fatigue capacity in mice [35,36], whilst limb grip strength is a key measure of muscular strength [37]. The plant-derived active ingredient combinations significantly enhanced limb grip strength in mice. Moreover, Formula 1 notably increased exhaustion swimming time, thereby improving endurance and strength levels in mice. The model used in this study primarily recapitulates hypobaric hypoxia as a single environmental stressor and does not fully replicate the integrated environmental load encountered under genuine high-altitude conditions. While this simplification helps clarify the causal relationship between hypobaric hypoxia and the intervention with plant bioactive compounds, caution is warranted when extrapolating the present findings to real-world high-altitude scenarios, where other environmental factors, such as temperature, may also exert influences.

Blood calcium levels reflect the body’s acid-base balance and assess the intensity of stress responses [38]. Changes in serum glucose levels in mice following exercise reflect the body’s energy metabolism status, hormonal regulatory capacity, and the immediate and adaptive effects of exercise on health [39]. Lactate dehydrogenase (LDH) is a key enzyme in lactate metabolism regulation, playing a crucial role during exercise; its activity level directly affects lactate production and removal rates, impacting muscle fatigue and exercise performance [40,41]. Furthermore, alterations in serum creatine kinase (CK) activity serve as a significant indicator of muscle damage and nutritional deficiency [42], whilst elevated urea levels can adversely affect muscle contraction and strength, thereby exacerbating fatigue [43]. Changes in serum creatinine levels are used to assess exercise intensity and muscular metabolic load, with higher exercise intensity and greater muscular load leading to a sharp increase in serum creatinine concentration [44]. Therefore, effectively clearing metabolites such as LDH, CK, urea, and serum creatinine, and restoring normal muscle tissue function, are key to alleviating post-exercise fatigue. The combination of plant bioactive compounds effectively maintains blood glucose levels before and after exercise, ameliorates glucose shortage following exercise under hypoxic conditions, significantly reduces post-exercise serum levels of LDH and CK, and decreases creatinine production. Consequently, it endows hypoxic mice with enhanced endurance performance and improved fatigue resistance.

HIF-1α is a key transcription factor that responds to hypoxic conditions and plays a crucial role in high-altitude hypoxia [45]. Hypoxic exposure activates the core adaptive pathway—the HIF-1 signalling pathway [46]. The activation of this pathway triggers a cascade of reactions that promote the production of erythropoietin (EPO) [47], thereby regulating erythrocytosis and enhancing the body’s oxygen-carrying capacity [48]. Hypoxic exposure also activates the body’s VEGF signalling pathway [49]. The activation of these two pathways promotes VEGF production, thereby facilitating angiogenesis and enhancing the body’s capacity to resist hypoxia [50,51]. Formula 1 significantly increased the protein level of HIF-1α and the content of VEGF, which amplified the hypoxic adaptation signal, activated corresponding pathways, elevated EPO levels, and enhanced the body’s hypoxia tolerance. In contrast, Formula 2 exhibited an extremely low level of HIF-1α protein, suggesting that it may not have activated the HIF-1 signal transduction pathway.

The levels of MDA [52], SOD [53], and GSH [54] reflect the body’s oxidative stress status. Exercise under high-altitude hypoxia exacerbates oxidative stress, potentially leading to a certain degree of oxidative damage [55]. Formula 1 significantly reduced MDA levels and increased the contents of SOD and GSH, thereby enhancing the body’s antioxidant capacity and mitigating oxidative stress damage induced by high-altitude hypoxic exercise. In contrast, Formula 2 showed only a significant effect on elevating SOD levels. Exercise in a high-altitude hypoxic environment prompts the body to transition to anaerobic energy production more rapidly [56]. Blood lactic acid (BLA) production exceeds intracellular buffering capacity, leading to a dramatic increase in BLA levels, which directly affects muscle performance [57,58,59]. Glycogen plays a key role in exercise performance; adequate glycogen reserves provide energy for sustained endurance exercise and support rapid energy release during high-intensity bursts [60]. When glycogen is depleted, and blood glucose levels cannot be maintained, hypoglycaemic symptoms may occur, resulting in fatigue and an inability to maintain exercise intensity [61]. The plant bioactive compound combinations Formula 1 and Formula 2 significantly reduced blood lactate levels and increased muscle glycogen storage, thereby enhancing resistance to exercise-induced fatigue in hypoxic mice.

SCFAs produced by gut microbes may provide energy by influencing lactate metabolism, increasing skeletal muscle glycogen synthesis, improving intestinal barrier function, and thus enhancing exercise performance [62,63,64]. SCFAs produced in the intestinal tract can enter the intramural circulation and reduce lactic acid accumulation in muscle by increasing the expression of lactate carriers MCT1 and MCT4 on muscle cell surfaces, promoting rapid translocation of lactic acid to surrounding tissues [62]. SCFAs activate the AMPK system, promote the expression of skeletal muscle glucose transporter GLUT4 protein, increase glucose uptake by muscle cells, and bind to G-protein-coupled receptors FFAR2 and FFAR3 to activate glycogen synthase, thereby increasing muscle glycogen content [65]. It is hypothesised that a combination of plant bioactive components can boost SCFA production, improve post-fatigue lactate metabolism in muscle, alter skeletal muscle metabolism, elevate glycogen levels, and consequently improve exercise performance.

In this study, it was found that after intervention with Plant Bioactive Component Combination 1, the serum levels of adenosine, choline, and isoleucine increased. The core issue in high-altitude hypoxic environments is inadequate oxygen supply, which restricts mitochondrial oxidative phosphorylation [66], leading to impaired ATP synthesis [67], accumulation of metabolic waste products, oxidative stress, and accelerated fatigue. Adenosine is one of the most potent endogenous vasodilators in the body [68]. It specifically dilates skeletal muscle microvessels by activating vascular endothelium through the A_2_A receptor [69]. Furthermore, adenosine enhances the stability and transcriptional activity of HIF-1α [70]. This action regulates the expression of genes related to EPO, VEGF, glycolytic enzymes, and mitochondrial remodelling. Consequently, it promotes increased red blood cell production, enhances blood oxygen-carrying capacity, stimulates angiogenesis in skeletal muscle microvessels, increases tissue perfusion reserve, remodels the metabolic enzyme profile, and ultimately improves glycolytic efficiency under hypoxic conditions [71]. Under hypoxic conditions, the decline in cerebral oxygen delivery disrupts the synthesis and metabolism of central neurotransmitters, leading to a rapid depletion of acetylcholine [72]. These neurochemical alterations directly manifest as diminished muscle contraction force, impaired motor coordination, and the premature onset of central fatigue/drowsiness. Given that choline is the exclusive biosynthetic precursor for acetylcholine, elevated choline levels may facilitate the restoration of acetylcholine synthesis, thereby potentially ameliorating deficits in muscle contractile function and strength performance [73]. Isoleucine can be oxidised directly in muscle for energy [74] and activates the key pathway regulating protein synthesis—the mTOR pathway [75]—thereby stimulating muscle protein synthesis and contributing to post-exercise muscle repair and growth [76]. Following intervention with Plant Bioactive Component Combination 1, the serum concentrations of the aforementioned active metabolites associated with enhanced hypoxic exercise performance were promoted in mice. Concomitantly, alterations were observed in metabolic pathways, including the “One carbon pool by folate” and “Biosynthesis of unsaturated fatty acids”. Furthermore, post-intervention faecal analysis revealed a decrease in the content of various amino acids, along with changes in the “Biosynthesis of unsaturated fatty acids” and “Tryptophan metabolism” pathways.

This study also found that intervention with Plant Bioactive Component Combination 2 increased the serum levels of L-arginine and phenylalanine. As the exclusive precursor of nitric oxide (NO), L-arginine predominantly mediates vasodilation [77]. It increases blood flow to working muscles and the heart, thereby enhancing tissue oxygen uptake and substrate supply despite low arterial partial pressure of oxygen, whilst simultaneously accelerating the clearance of lactate and H^+^ [78,79]. Phenylalanine contributes to delaying fatigue onset, maintaining strength and endurance output, and enhancing tolerance to hypoxic stress [80] by sustaining central motor drive and sympathetic tone [81], protecting myofibrillar structure [82], and improving mitochondrial oxidative efficiency [83]. Following intervention with Plant Bioactive Component Combination 2, an increase in serum concentrations of the aforementioned active metabolites was observed, associated with enhanced hypoxic exercise performance in mice. Concurrently, alterations were noted in the “Arginine and proline metabolism” and “Phenylalanine, tyrosine and tryptophan biosynthesis” pathways. Furthermore, analysis of faecal samples revealed changes in the “Arginine biosynthesis” and “Arachidonic acid metabolism” pathways post-intervention. Therefore, it is hypothesised that the plant bioactive component combination may regulate exercise performance under hypoxic conditions by modulating the body’s energy metabolism and activating relevant pathways.

## 5. Conclusions

In this study, we observed that supplementation with plant bioactive components effectively enhanced exercise performance in hypoxic mice. Furthermore, other studies have shown that plant bioactive components, such as kaempferol [84] and lycopene [85], can also affect exercise performance under hypoxic conditions and enhance the body’s hypoxic adaptation. The plant bioactive component combination improved the exercise performance of hypoxic mice by reducing LA, LDH, and CK levels, increasing liver and skeletal muscle glycogen content, and maintaining post-exercise blood glucose levels. These beneficial effects are likely mediated through influencing serum and faecal metabolites, particularly SCFAs and amino acids. This research provides novel insights for developing functional interventions to promote exercise performance in hypoxic environments. Although this work provides practical implications for hypoxia-targeted functional interventions, as a preclinical study, it has unavoidable limitations. The application scenarios should be clearly delimited.

## Data Availability

The original contributions presented in the study are included in the article. Further inquiries can be directed to the corresponding author.

## Supplementary Materials

The following supporting information can be downloaded at: https://www.mdpi.com/article/10.3390/nu18091349/s1, Section S1: Search strategy; Section S2: Evaluation of inconsistency and heterogeneity; Section S3: Funnel plots; Section S4: SUCRA and cumulative probability plots; Section S5: league table of Summary Estimates; Section S6: SYRCLE risk of bias assessment results.

## Abbreviations

The following abbreviations are used in this manuscript:

SCFA	short-chain fatty acid
LA	lactic acid
LDH	lactate dehydrogenase
CK	creatine kinase
PAH	pulmonary arterial hypertension
HIF-1α	hypoxia-inducible factor-1α
EPO	erythropoietin
MDA	malondialdehyde
SOD	superoxide dismutase
GSH	glutathione
SUCRA	surface under the cumulative ranking curve
APS	astragalus polysaccharide
GP	gypenoside
RBC	red blood cells
HGB	haemoglobin
HCT	haematocrit
MCV	mean corpuscular volume
SCR	serum creatinine
VEGF	vascular endothelial growth factor
VD3	vitamin D3
KEGG	Kyoto Encyclopedia of Genes and Genomes
