# Supplementary material for: Enhancing Exercise Performance Under Hypoxia: A Network Meta-Analysis and Animal Experimental Validation of Plant Bioactive Compounds
Source: Nutrients. 2026 Apr 24;18(9):1349. doi: 10.3390/nu18091349 (PMC13164976; doi:10.3390/nu18091349)
Supplement: Supplementary file 1 [file nutrients-18-01349-s001.zip › nutrients-4229421-supplementary.pdf]

Supplementary Materials

Enhancing Exercise Performance Under Hypoxia: A Network Meta-Analysis and Animal Experimental Validation of Plant Bioactive Compounds

Table of contents

Sections S1: Search strategy.. 2

Sections S2: Evaluation of inconsistency and heterogeneity 3

Sections S3: Funnel plots 13

Sections S4: SUCRA and cumulative probability plots 18

Sections S5: league table of Summary Estimates.. 27

Sections S6: SYRCLE risk of bias assessment results 39

Sections S1: Search strategy

(1) Search strategy of PubMed

((((((((Hypoxia[MeSH Terms]) OR (Deficiency, Oxygen[Title/Abstract])) OR (Deficiencies, Oxygen[Title/Abstract])) OR (Oxygen Deficiencies[Title/Abstract])) OR (Oxygen Deficiency[Title/Abstract])) OR (Anoxemia[Title/Abstract])) OR (Hypoxemia[Title/Abstract])) OR (Anoxia[Title/Abstract])) AND ((((((((((Alleviate[Title/Abstract]) OR (Ameliorate[Title/Abstract])) OR (Help[Title/Abstract])) OR (Allay[Title/Abstract])) OR (Ease[Title/Abstract])) OR (Relieve[Title/Abstract])) OR (Dull[Title/Abstract])) OR (Absorb[Title/Abstract])) OR (Deaden[Title/Abstract])) OR (Soothe[Title/Abstract])) OR (Alleviation[Title/Abstract]))) AND (("2019/01/01"[Date - Publication] : "2024/10/01"[Date - Publication]))

(2) Search strategy of Web of Science

((((((((TS=(Hypoxia)) OR TS=(Deficiency, Oxygen)) OR TS=(Deficiencies, Oxygen)) OR TS=(Oxygen Deficiencies)) OR TS=(Oxygen Deficiency)) OR TS=(Anoxemia)) OR TS=(Hypoxemia)) OR TS=(Anoxia)) AND ((((((((((TS=(Alleviate)) OR TS=(Ameliorate)) OR TS=(Help)) OR TS=(Allay)) OR TS=(Ease)) OR TS=(Relieve)) OR TS=(Dull)) OR TS=(Absorb)) OR TS=(Deaden)) OR TS=(Soothe)) OR TS=(Alleviation))) AND DOP=(2019-01-01/2024-10-01)

Sections S2: Evaluation of inconsistency and heterogeneity

Table S1: Global consistency

**Experimental outcome**	**Chi square**	**P value**
PAH	0	1
HIF-1α	518.78	0
EPO	8.47	0.0036
MDA	5.86	0.0533
SOD	429.76	0
GSH	0	1
LA	6.27	0.1797
Glycogen	0.01	0.9966
LDH	18.05	0.0001

Abbreviations: PAH, pulmonary arterial hypertension; HIF-1α, hypoxia inducible factor-1α; EPO, erythropoietin; MDA, malondialdehyde; SOD, superoxide dismutase; GSH, glutathione; LA, lactic acid; LDH, lactate dehydrogenase.

Table S1.1: Analysis results of PAH. Inconsistency test between direct and indirect intervention comparisons in different intervention comparisons.

Comparison	Direct		Indirect		Difference		
	Coef.	Std. Err.	Coef.	Std. Err.	Coef.	Std. Err.	P>z
Control V.S. Luteolin	-7.1	6.55786	-22.99132	14.72877	15.89132	16.14467	0.325
Control V.S. Astragaloside IV	-18.1	2.565698	4.181694	5.420722	-22.28169	6.163492	0
Control V.S. Paeoniflorin	-7.2	8.952742	-13.17235	20.04316	5.972347	21.9782	0.786
Luteolin V.S. Sildenafil	2.7	6.535712	-13.19132	14.75827	15.89132	16.14467	0.325
Sildenafil V.S. Astragaloside IV	-1	2.399084	-23.28169	5.644981	22.28169	6.16349	0
Sildenafil V.S. Paeoniflorin	0.5	8.93607	6.472346	20.06551	-5.972346	21.97823	0.786

Table S1.2: Analysis results of HIF-1α. Inconsistency test between direct and indirect intervention comparisons in different intervention comparisons.

Comparison	Direct		Indirect		Difference		
	Coef.	Std. Err.	Coef.	Std. Err.	Coef.	Std. Err.	P>z
Control V.S. Rhodiola	-5.288331	2.734537	6.742273	11.23301	-12.0306	11.72966	0.305
Control V.S. Acetazolamide	-8.789458	4.716301	-4.38995	12.58101	-4.399508	13.51425	0.745
Control V.S. Ginsenoside	-7.166695	4.43693	5.206657	12.27188	-12.37335	13.53544	0.361
Control V.S. L.delbrueckii	-1.991853	5.260157	-23.77417	13.92566	21.78232	14.90505	0.144
Control V.S. DSP	-3.145449	6.335878	-3.143961	12.6997	-0.0014878	14.21856	1
Control V.S. Turnip	7.903587	4.396972	-13.09549	8.367693	20.99908	9.467242	0.027
Rhodiola V.S. Acetazolamide	-5.61808	6.331906	-1.520768	6.681436	-4.097312	9.094976	0.652
Rhodiola V.S. Ginsenoside	1.263561	6.184552	-3.471242	6.66655	4.734803	9.150795	0.605
Rhodiola V.S. DSP	1.405413	6.323495	1.403926	12.7182	0.0014876	14.21856	1
Rhodiola V.S. Turnip	3.719335	4.183316	24.71841	8.690108	-20.99908	9.467242	0.027
Acetazolamide V.S. L.delbrueckii	0.4659839	5.24277	22.2483	13.94532	-21.78232	14.90505	0.144

Abbreviations: DSP, compound danshen dripping pills.

Table S1.3: Analysis results of MDA. Inconsistency test between direct and indirect intervention comparisons in different intervention comparisons.

Comparison	Direct		Indirect		Difference		
	Coef.	Std. Err.	Coef.	Std. Err.	Coef.	Std. Err.	P>z
Control V.S. Rhodiola	-3.858263	1.117662	-2.050251	3.264771	-1.808012	3.451855	0.6
Control V.S. Synonyms	-1.817731	0.6795799	-8.654535	3.292805	6.836803	3.363776	0.042
Control V.S. DSP	-0.1597096	1.816078	-4.36141	3.608133	4.2017	4.040832	0.298
Control V.S. EGCG	-2.192374	2.021241	-1.945133	4.017463	-0.2472416	4.515585	0.956
Control V.S. Acteoside	-2.135565	1.915986	-6.912711	4.054373	4.777146	4.494622	0.288
Control V.S. Echinacoside	-2.000403	1.913855	-6.777549	4.05513	4.777146	4.494623	0.288
Control V.S. Ginsenoside	-4.662823	1.557395	-5.458629	4.421801	0.7958067	4.722683	0.866
Control V.S. L.delbrueckii	-2.808273	1.978439	1.130573	4.24106	-3.938846	4.778431	0.41
Control V.S. Kaempferol	-3.70261	2.02767	-1.783343	5.131397	-1.919266	5.63601	0.733
Control V.S. Puerarin	-0.3982822	2.006261	-1.652186	4.566313	1.253904	4.992298	0.802
Control V.S. Polygonatum	-2.320178	2.063831	-3.012237	4.038258	0.6920598	4.55807	0.879
Control V.S. Crocetin	-1.206784	1.969284	-3.619566	4.455143	2.412782	4.875449	0.621
Control V.S. Turnip	-12.60144	2.756143	11.95198	4.207435	-24.55343	6.452307	0
Rhodiola V.S. Synonyms	-1.049155	1.584845	2.645559	1.358844	-3.694714	2.084188	0.076
Rhodiola V.S. Acteoside	-0.3243897	1.898939	4.452756	4.078363	-4.777146	4.494623	0.288
Rhodiola V.S. Echinacoside	-0.1892273	1.898672	4.587919	4.076485	-4.777146	4.494623	0.288
Rhodiola V.S. Ginsenoside	-0.9673267	2.019421	-1.157482	2.426269	0.1901554	3.140608	0.952
Rhodiola V.S. Turnip	2.295612	1.345041	-22.25781	5.921491	24.55343	6.452306	0
Synonyms V.S. DSP	0.2916437	1.816877	4.493344	3.606927	-4.2017	4.040832	0.298
Synonyms V.S.	0.080602	2.002156	-0.1666396	4.046031	0.2472416	4.515585	0.956

EGCG							
Synonyms V.S. Polygonatum	-0.4274011	2.024721	0.2646587	4.097223	-0.6920598	4.55807	0.879
Ginsenoside V.S. Kaempferol	1.586833	1.954715	-0.3324343	5.215632	1.919267	5.636008	0.733
L.delbrueckii V.S. Acetazolamide	-0.3008864	1.88006	3.63796	4.373258	-3.938846	4.778431	0.41
Acetazolamide V.S. Puerarin	0.9309246	2.010837	2.184829	4.560271	-1.253904	4.992298	0.802
Acetazolamide V.S. Crocetin	-0.2552813	1.960093	2.1575	4.467286	-2.412782	4.875449	0.621

Abbreviations: EGCG, (-)-epigallocatechin.

Table S1.4: Analysis results of SOD. Inconsistency test between direct and indirect intervention comparisons in different intervention comparisons.

Comparison	Direct		Indirect		Difference		
	Coef.	Std. Err.	Coef.	Std. Err.	Coef.	Std. Err.	P>z
Control V.S. Rhodiola	3.583748	1.05226	4.972567	5.245552	-1.388819	5.349975	0.795
Control V.S. Synonyms	2.716816	1.167175	6.073693	4.036445	-3.356877	4.202683	0.424
Control V.S. DSP	1.817472	1.894131	3.273407	5.300623	-1.455934	5.646866	0.797
Control V.S. CRB	-2.051181	2.05	8.885612	3.95649	-10.93679	4.457744	0.014
Control V.S. GP	5.576806	2.666231	1.027733	4.930176	4.549073	5.747193	0.429
Control V.S. Acteoside	1.870623	2.592019	5.489604	5.001562	-3.61898	5.644564	0.521
Control V.S. Echinacoside	1.757252	2.590861	5.376232	5.00235	-3.61898	5.644564	0.521
Control V.S. Ginsenoside	4.648523	2.760191	2.742126	5.094489	1.906397	5.921639	0.748
Control V.S. L.delbrueckii	1.147717	2.63578	-2.445788	6.942102	3.593505	7.451649	0.63
Control V.S. Polygonatum	2.384288	2.664433	1.95774	5.263566	0.4265477	5.948115	0.943
Control V.S. Crocetin	1.505726	2.606157	5.099231	6.983787	-3.593505	7.451648	0.63
Control V.S. Turnip	5.874107	2.496201	-7.130216	4.953341	13.00432	6.042831	0.031
Rhodiola V.S. Synonyms	0.1371454	2.620347	-1.005348	1.742935	1.142494	3.146991	0.717
Rhodiola V.S. CRB	-0.9322086	2.033817	-11.869	3.981466	10.93679	4.457744	0.014
Rhodiola V.S. GP	1.77E-12	2.510257	4.549073	5.169994	-4.549073	5.747193	0.429
Rhodiola V.S. Acteoside	-0.2267422	2.582293	-3.845723	5.016633	3.61898	5.644565	0.521
Rhodiola V.S. Echinacoside	-0.3401133	2.582475	-3.959094	5.015343	3.61898	5.644565	0.521
Rhodiola V.S. Ginsenoside	0.1936885	2.60874	2.100085	5.328527	-1.906397	5.921638	0.748
Rhodiola V.S. Turnip	-3.554082	2.325187	9.450241	5.197035	-13.00432	6.042831	0.031
Synonyms V.S. DSP	-0.8046435	2.660076	-1.208421	2.815644	0.4037777	3.880059	0.917
Synonyms V.S. Polygonatum	-0.7712389	2.6349	-0.3446912	5.307979	-0.4265477	5.948115	0.943
Acetazolamide V.S. L.delbrueckii	-1.157125	2.635981	2.43638	6.941874	-3.593505	7.451648	0.63
Acetazolamide V.S. Crocetin	0.997636	2.599815	-2.595869	6.990876	3.593505	7.451649	0.63

Abbreviations: GP, Gypenosides; CRB, Citrus reticulata Blanco.

Table S1.5: Analysis results of GSH. Inconsistency test between direct and indirect intervention comparisons in different intervention comparisons.

Comparison	Direct		Indirect		Difference		
	Coef.	Std. Err.	Coef.	Std. Err.	Coef.	Std. Err.	P>z
Control V.S. Rhodiola	5.060638	1.612524	6.003431	7.178299	-0.9427925	7.371736	0.898
Control V.S. Synonyms	3.381505	2.680164	6.083648	7.39711	-2.702143	7.890703	0.732
Control V.S. CRB	1.435752	3.26886	11.52882	6.434925	-10.09307	7.218727	0.162
Control V.S. Acteoside	1.257969	3.351836	10.59523	6.599397	-9.337263	7.404587	0.207
Control V.S. Echinacoside	0.9434766	3.350328	10.28074	6.600284	-9.337263	7.404587	0.207
Control V.S. Ginsenoside	5.445807	2.734442	2.415471	7.354775	3.030335	7.941444	0.703
Control V.S. Puerarin	4.881461	3.410171	-6.907422	8.904023	11.78888	9.653382	0.222
Control V.S. Turnip	5.51396	3.642483	-1.502057	7.122285	7.016017	8.270302	0.396
Control V.S. Crocetin	1.38076	3.32568	13.16964	9.05968	-11.78888	9.653383	0.222
Rhodiola V.S. Synonyms	-0.8314275	3.752661	-2.038215	3.94347	1.206787	5.448903	0.825
Rhodiola V.S. CRB	0.5743009	3.263613	-9.518768	6.442911	10.09307	7.218728	0.162
Rhodiola V.S. Acteoside	0.0698872	3.348399	-9.267376	6.604629	9.337263	7.404587	0.207
Rhodiola V.S. Echinacoside	-0.244605	3.348519	-9.581868	6.603038	9.337263	7.404587	0.207
Rhodiola V.S. Ginsenoside	-0.9114488	3.697327	0.9584327	4.000414	-1.869882	5.461491	0.732
Rhodiola V.S. Turnip	-2.539243	3.516342	4.476774	7.309995	-7.016017	8.270302	0.396
Acetazolamide V.S. Puerarin	-2.051034	3.334646	9.737849	8.989428	-11.78888	9.653382	0.222
Acetazolamide V.S. Crocetin	0.3427066	3.31869	-11.44618	9.067366	11.78888	9.653382	0.222

Table S1.6: Analysis results of LA. Inconsistency test between direct and indirect intervention comparisons in different intervention comparisons.

Comparison	Direct		Indirect		Difference		
	Coef.	Std. Err.	Coef.	Std. Err.	Coef.	Std. Err.	P>z
Control V.S. Rhodiola	-1.505203	0.2752755	-0.001607	1.184848	-1.503596	1.222142	0.219
Control V.S. Synonyms	-0.8788105	0.2696347	-3.192937	1.045339	2.314127	1.081834	0.032
Control V.S. DSP	-0.7840414	0.6872421	-2.929001	1.312468	2.14496	1.46857	0.144
Control V.S. CRB	-1.86415	0.6265883	-0.9696915	1.222168	-0.8944582	1.417537	0.528
Control V.S. Acteoside	-2.423182	0.5694049	-0.7691867	1.057671	-1.653996	1.250022	0.186
Control V.S. Echinacoside	-1.600215	0.5252543	0.0537809	1.081293	-1.653996	1.250022	0.186
Control V.S. Curcumin	-1.625495	0.6097905	-2.471175	1.232393	0.8456796	1.370771	0.537
Rhodiola V.S. Synonyms	-0.4084137	0.5036921	0.9707386	0.4275128	-1.379152	0.6603003	0.037
Rhodiola V.S. CRB	-0.0981131	0.5692196	-0.9925713	1.303649	0.8944582	1.417537	0.528
Rhodiola V.S. Acteoside	-0.3657634	0.4903759	-2.019759	1.170439	1.653996	1.250022	0.186
Rhodiola V.S. Echinacoside	0.4572042	0.4914946	-1.196791	1.127905	1.653996	1.250022	0.186
Synonyms V.S. DSP	-0.642005	0.6800288	1.502955	1.323692	-2.14496	1.46857	0.144
Synonyms V.S. Curcumin	-0.9030528	0.579381	-0.0573732	1.275649	-0.8456796	1.370771	0.537

Table S1.7: Analysis results of Glycogen. Inconsistency test between direct and indirect intervention comparisons in different intervention comparisons.

Comparison	Direct		Indirect		Difference		
	Coef.	Std. Err.	Coef.	Std. Err.	Coef.	Std. Err.	P>z
Control V.S. Rhodiola	0.0933333	0.0238048	0.1032649	0.071383	-0.0099316	0.0716161	0.89
Control V.S. Synonyms	0.0390009	0.0184312	-0.0396157	0.0948899	0.0786166	0.0980057	0.422
Control V.S. DSP	0.17	0.1029563	0.18136	0.1248871	-0.01136	0.1224614	0.926
Control V.S. Acteoside	0.14	0.0342053	0.2186166	0.0966184	-0.0786166	0.0980057	0.422
Control V.S. Echinacoside	0.03	0.0368782	0.1086166	0.0975967	-0.0786166	0.0980057	0.422
Rhodiola V.S. Synonyms	-0.0708144	0.0349409	-0.0433035	0.0394671	-0.0275109	0.0521356	0.598
Rhodiola V.S. Acteoside	0.06	0.0360555	-0.0186166	0.0945787	0.0786166	0.0980057	0.422
Rhodiola V.S. Echinacoside	-0.05	0.0386005	-0.1286166	0.0955778	0.0786166	0.0980057	0.422
Synonyms V.S. DSP	0.14	0.0948683	0.12864	0.1428174	0.01136	0.1224614	0.926

Table S1.8: Analysis results of LDH. Inconsistency test between direct and indirect intervention comparisons in different intervention comparisons.

Comparison	Direct		Indirect		Difference		
	Coef.	Std. Err.	Coef.	Std. Err.	Coef.	Std. Err.	P>z
Control V.S. DSP	-0.4523644	1.799367	-4.034917	4.041927	3.582552	4.431792	0.419
Control V.S. EGCG	-2.327523	2.067521	-2.612519	4.66661	0.2849961	5.117519	0.956
Control V.S. Acteoside	-1.731715	1.096736	-8.697795	3.445891	6.966079	3.631922	0.055
Control V.S. Echinacoside	-1.24301	1.087019	-8.20909	3.451054	6.966079	3.63192	0.055
Control V.S. Polygonatum	-2.226816	1.724329	1.760208	3.86464	-3.987024	4.309772	0.355
Rhodiola V.S. Acteoside	0.2602884	1.077055	7.226368	3.464465	-6.96608	3.631922	0.055
Rhodiola V.S. Echinacoside	0.7489933	1.080392	7.715073	3.457293	-6.96608	3.631922	0.055
Synonyms V.S. DSP	0.5216152	1.80028	4.104167	4.040708	-3.582552	4.431793	0.419
Synonyms V.S. EGCG	-0.243174	2.046687	0.0418221	4.694082	-0.2849961	5.11752	0.956
Synonyms V.S. Polygonatum	1.31613	1.695318	-2.670895	3.902957	3.987024	4.309774	0.355

Sections S3: Funnel plots

Fig. S1.1: Funnel plot of PAH

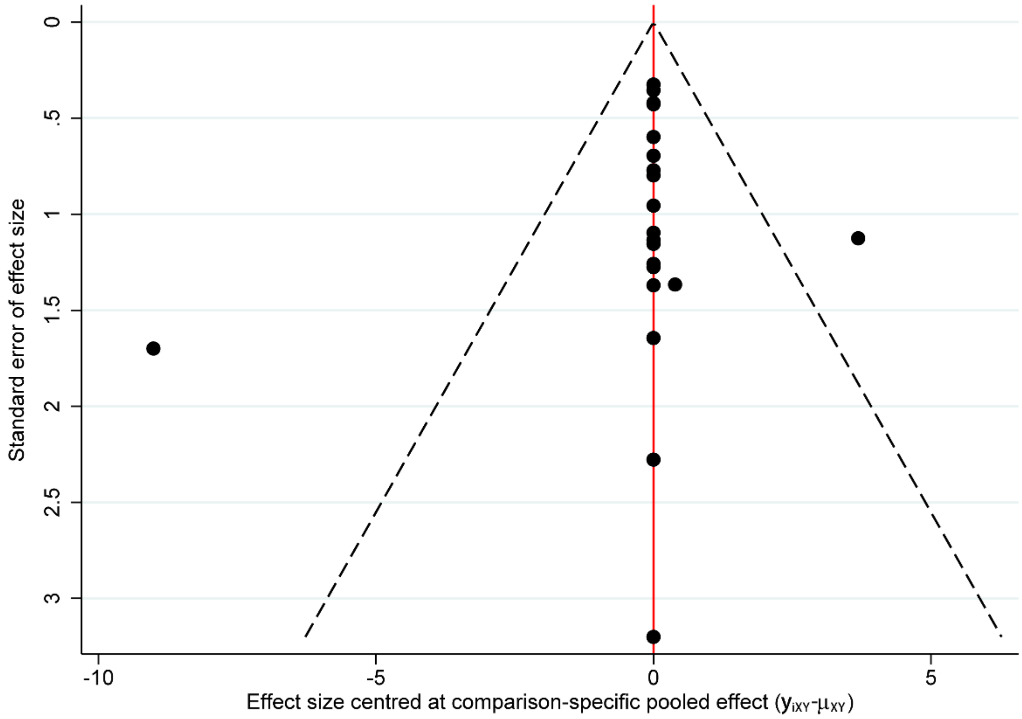

Fig. S1.2: Funnel plot of HIF-1α

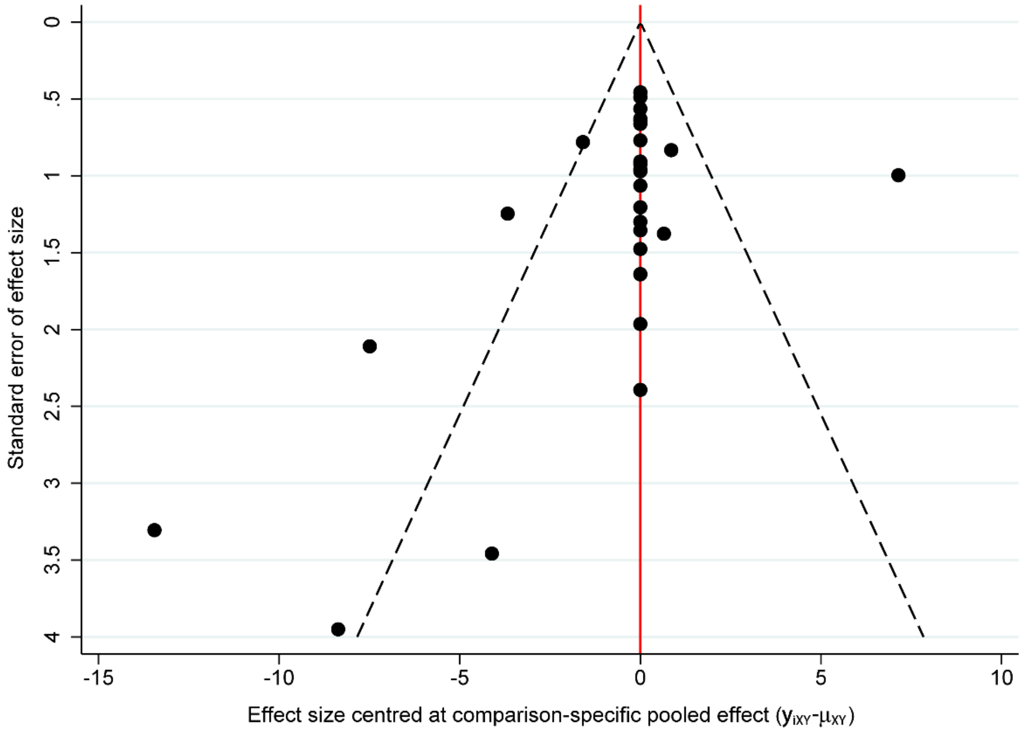

Fig. S1.3: Funnel plot of EPO

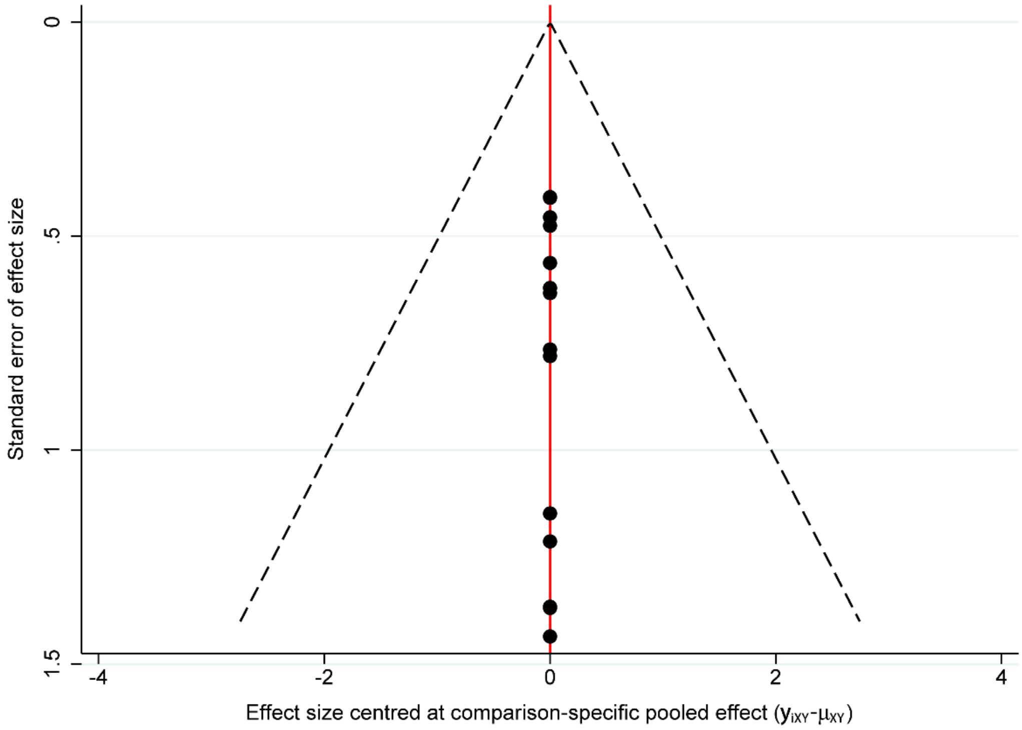

Fig. S1.4: Funnel plot of MDA

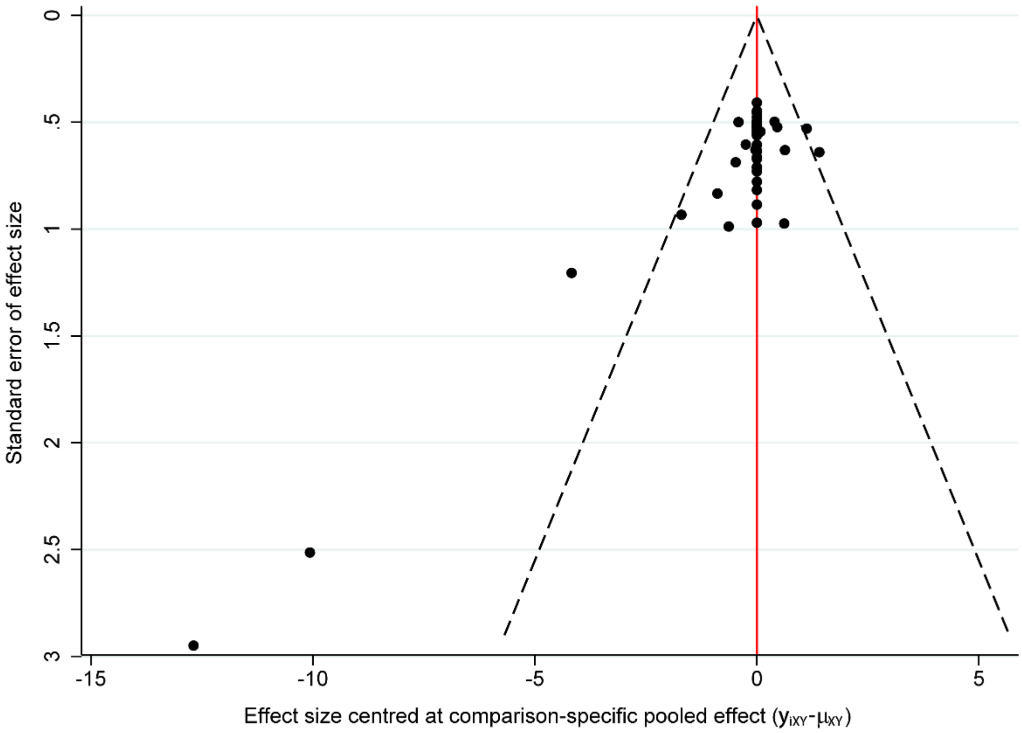

Fig. S1.5: Funnel plot of SOD

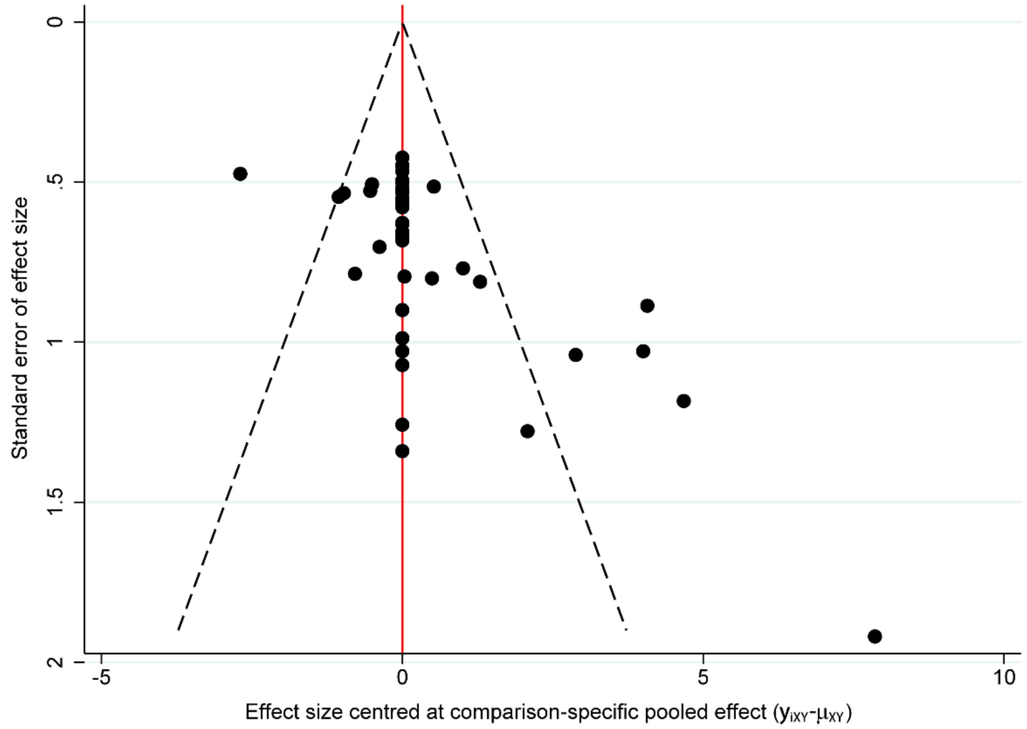

Fig. S1.6: Funnel plot of GSH

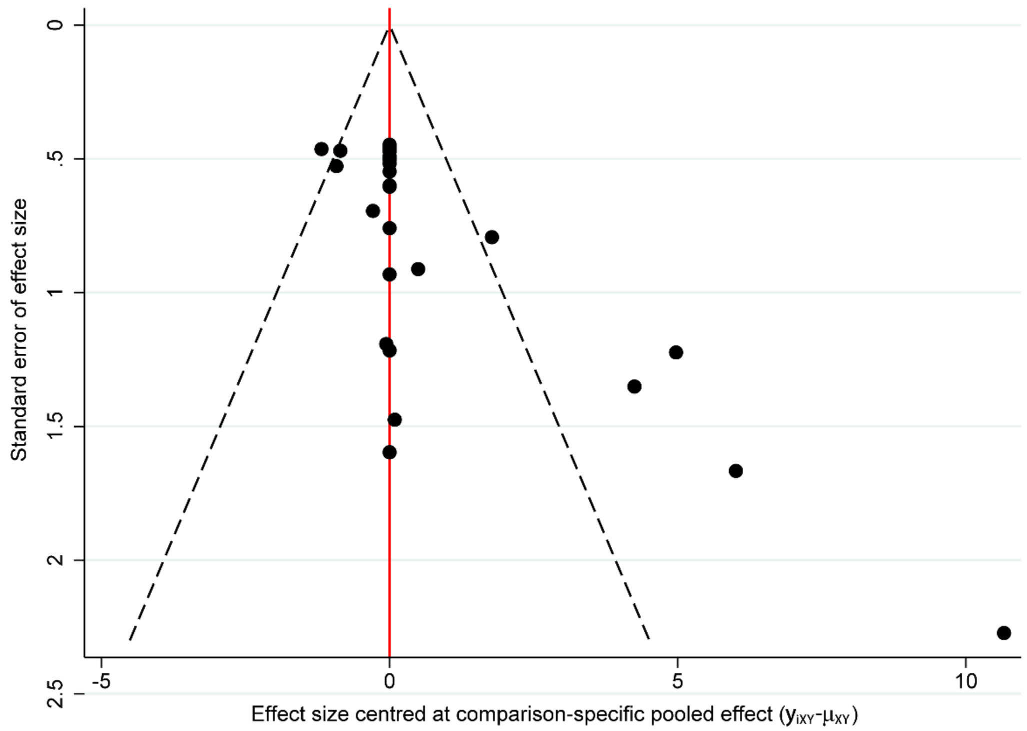

Fig. S1.7: Funnel plot of LA

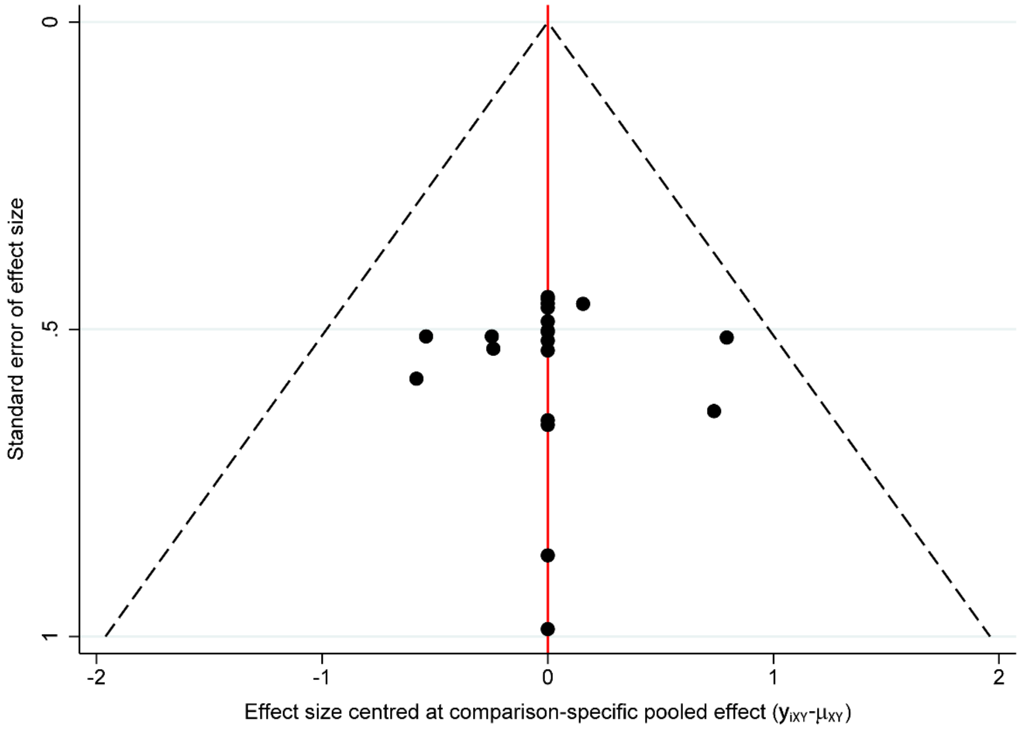

Fig. S1.8: Funnel plot of Glycogen

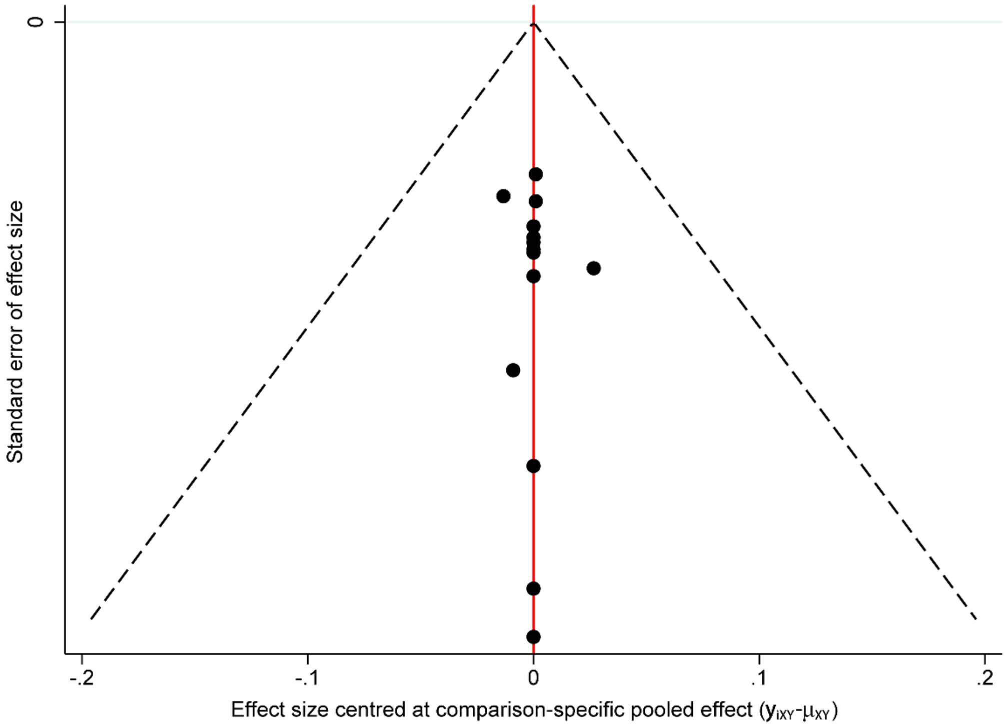

Fig. S1.9: Funnel plot of LDH

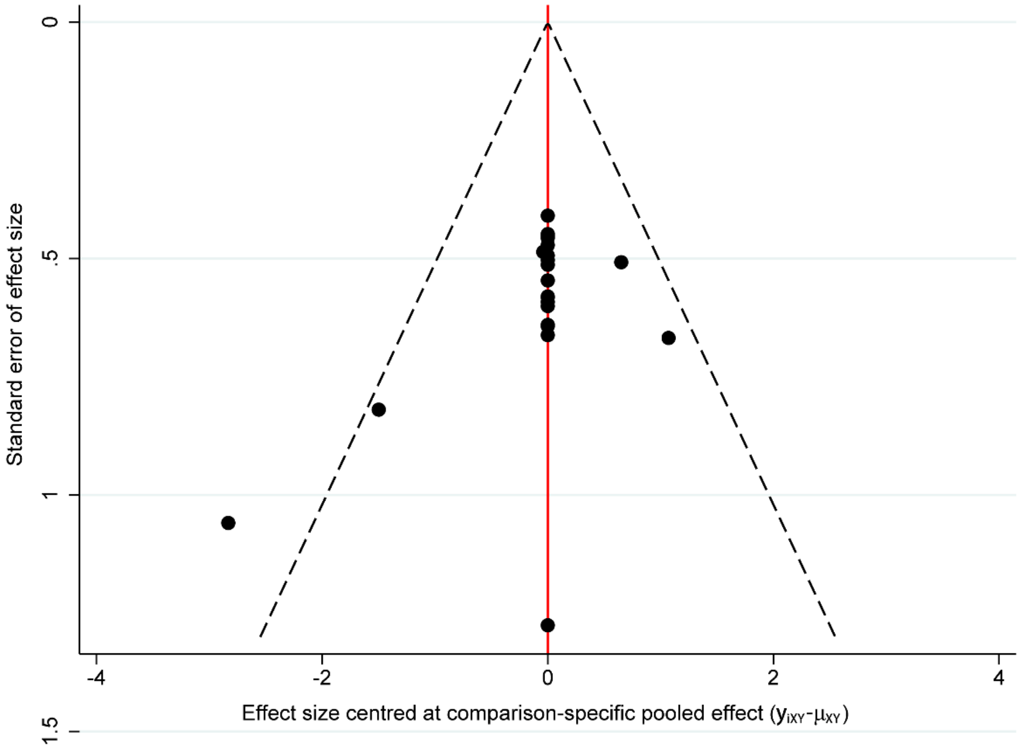

Standard error of effect size
0
.5
1
1.5
-4
-2
0
2
4
Effect size centred at comparison-specific pooled effect (yiXY-μXY)

Sections S4: SUCRA and cumulative probability plots

Table S2.1: SUCRA of the effects of different active components for PAH.

Treatment	SUCRA	PrBest	MeanRank
**Puerarin**	97.1	77.2	1.4
**Nifedipine**	71	5.2	5.3
**AstragalosideIV**	69.2	3.2	5.6
**Ginsenoside**	66.8	4	6
**Lycopene**	63.2	3.7	6.5
**Catechin**	59.2	2.8	7.1
**TanshinoneIIA**	52.5	1.1	8.1
**Luteolin**	51.6	1	8.3
**Sildenafil**	50.8	0	8.4
**APS**	49.8	0.9	8.5
**Paeoniflorin**	45	0.5	9.3
**VD3**	40.7	0.3	9.9
**NGFS**	37.4	0	10.4
**Acetazolamide**	18.3	0	13.3
**Rhodiola**	16.3	0	13.6
**control**	11.1	0	14.3

Abbreviations: SUCRA, surface under the cumulative ranking curve; APS, astragalus polysaccharide; VD3, vitamin D3; NGFS, *Nigellalandulifera Preynet* Sint.

Table S2.2: SUCRA of the effects of different active components for HIF-1α.

Treatment	SUCRA	PrBest	MeanRank
**APS**	85.6	37	3.4
**ASF**	79	20	4.6
**Polygonatum**	77.4	18.2	4.8
**Acetazolamide**	69	3.4	6.3
**Lycopene**	60	5.7	7.8
**Synonyms**	56.8	3.2	8.3
**Rutin**	54.7	2.5	8.7
**Ginsenoside**	53.7	0.9	8.9
**AstragalosideIV**	50.6	3.2	9.4
**L.delbrueckii**	50.4	2	9.4
**Rhodiola**	49.6	0	9.6
**DSP**	42.4	0.9	10.8
**Luteolin**	38.4	1.1	11.5
**LGG**	36.7	0.8	11.8
**Curcumin**	34.1	0.6	12.2
**L.caseiATCC393**	25.4	0.3	13.7
**control**	21.8	0	14.3
**Turnip**	14.5	0	15.5

Abbreviations: ASF, *Aster souliei* Franchon; LGG, stachyose in combination with *L. rhamnosus* GG.

Table S2.3: SUCRA of the effects of different active components for EPO.

Treatment	SUCRA	PrBest	MeanRank
**Nifedipine**	97	74.2	1.3
**AstragalosideIV**	81.3	0	2.7
**Arginine**	71.5	21.9	3.6
**Rhodiola**	59	0	4.7
**Ginsenoside**	51.6	0	5.4
**Acetazolamide**	44.1	3.9	6
**L.delbrueckii**	43.6	0	6.1
**EGCG**	22	0	8
**Synonyms**	20.5	0	8.2
**control**	9.5	0	9.1

Table S2.4: SUCRA of the effects of different active components for MDA.

Treatment	SUCRA	PrBest	MeanRank
**Ginsenoside**	87.3	20.3	3.9
**Hyperoside**	81.3	28.4	5.3
**MAP**	69.5	9.9	8
**Rhodiola**	67.7	0.5	8.4
**Paeoniflorin**	66.2	7.6	8.8
**Kaempferol**	66.1	4.7	8.8
**Lycopene**	63.2	8.2	9.5
**L.caseiATCC393**	63	6.2	9.5
**Acteoside**	59.6	2.6	10.3
**Turnip**	59.1	1.4	10.4
**Echinacoside**	56.1	2.6	11.1
**Polygonatum**	51.5	1.6	12.2
**ASF**	49.6	2.2	12.6
**EGCG**	45.5	1	13.5
**Synonyms**	44.4	0	13.8
**L.delbrueckii**	43.6	1.1	14
**Rutin**	37.9	0.6	15.3
**Acetazolamide**	36.6	0	15.6
**Crocetin**	36.1	0.5	15.7
**LGG**	33.2	0.4	16.4
**Atorvastatin**	29.8	0.1	17.1
**DSP**	25.6	0.2	18.1
**Puerarin**	20	0	19.4
**control**	7.5	0	22.3

Abbreviations: MAP, cortex mori polysaccharide.

Table S2.5: SUCRA of the effects of different active components for SOD.

Treatment	SUCRA	PrBest	MeanRank
**GP**	74.2	10	7.2
**Ginsenoside**	71.6	8	7.8
**Rhodiola**	68.7	0.2	8.5
**UrsolicAcid**	67.5	9.9	8.8
**Atorvastatin**	62.9	5.8	9.9
**Synonyms**	59.9	0.1	10.6
**Turnip**	57.2	0.7	11.3
**Acteoside**	53.7	1.7	12.1
**Echinacoside**	52.3	1.4	12.4
**Hyperoside**	49.6	2.4	13.1
**Polygonatum**	49.6	1	13.1
**Crocetin**	45.9	1	14
**DSP**	45.7	0.2	14
**Kaempferol**	44.8	1.2	14.2
**LGG**	44.5	1.2	14.3
**Paeoniflorin**	40.3	0.9	15.3
**L.caseiATCC393**	38.8	0.7	15.7
**Acetazolamide**	38	0.2	15.9
**MAP**	34.2	0.2	16.8
**L.delbrueckii**	31.8	0.3	17.4
**CRB**	27	0.1	18.5
**control**	18.2	0	20.6
**Cartialgenous**	6.3	0	23.5

Table S2.6: SUCRA of the effects of different active components for GSH.

Treatment	SUCRA	PrBest	MeanRank
**Rhodiola**	69.3	5.1	4.4
**Ginsenoside**	66.4	13.5	4.7
**UrsolicAcid**	63.9	24.7	5
**Turnip**	55.2	11.4	5.9
**Acetazolamide**	53.9	5.7	6.1
**Synonyms**	51.7	4.8	6.3
**CRB**	49.8	9.1	6.5
**Puerarin**	47.6	8.2	6.8
**Acteoside**	46.1	6.4	6.9
**Echinacoside**	43.2	5.1	7.2
**Crocetin**	42.6	6	7.3
**control**	10.4	0	10.9

Table S2.7: SUCRA of the effects of different active components for LA.

Treatment	SUCRA	PrBest	MeanRank
**Moringa**	93.9	67.6	1.5
**Resveratrol**	79	25.9	2.9
**Acteoside**	68.6	3	3.8
**Curcumin**	59.1	1.7	4.7
**CRB**	55.1	1.2	5
**Rhodiola**	43.5	0.1	6.1
**DSP**	38.1	0.4	6.6
**Echinacoside**	36.6	0.1	6.7
**Synonyms**	25.5	0	7.7
**control**	0.6	0	9.9

Table S2.8: SUCRA of the effects of different active components for LDH.

Treatment	SUCRA	PrBest	MeanRank
**VD3**	96.9	82.9	1.4
**Rhodiola**	75.2	4	4.2
**MAP**	63.3	3.6	5.8
**Rutin**	62	3.6	5.9
**Acteoside**	57.5	1.6	6.5
**EGCG**	55.9	1.6	6.7
**Synonyms**	53.1	0.1	7.1
**Echinacoside**	48.9	0.7	7.6
**Hyperoside**	45.1	1.1	8.1
**Polygonatum**	40.6	0.5	8.7
**Atorvastatin**	33.2	0.2	9.7
**DSP**	32.9	0.2	9.7
**ASF**	24.2	0	10.9
**control**	11.2	0	12.5

Table S2.9: SUCRA of the effects of different active components for Glycogen.

Treatment	SUCRA	PrBest	MeanRank
**Moringa**	99.9	99.7	1
**Acteoside**	72.1	0	2.7
**DSP**	69.7	0.3	2.8
**Rhodiola**	53.5	0	3.8
**Synonyms**	26.1	0	5.4
**Echinacoside**	24.9	0	5.5
**control**	3.8	0	6.8

Sections S5: league table of Summary Estimates

League table of summary estimates for different active components on enhancing exercise performance under hypoxic conditions derived from network meta-analysis of 49 studies.

Table S3.1: PAH

The columns represent the comparison of the row active components class to the column active components class. The rows represent the comparison of the row active components class to the column active components class. The effect estimates are expressed as mean difference and 95% confidence interval. For example, the mean difference in PAH for Puerarin compared to Nifedipine is -12.57 (95% confidence interval -30.40 to 5.26). Mean difference <0 favors the active componentsg in the column, and mean difference >0 favors the active components in the row.

**Puerarin**						
-12.57 (-30.40,5.26)	**Nifedipine**					
-13.10 (-30.32,4.12)	-0.53 (-17.76,16.71)	**AstragalosideIV**				
-13.71 (-31.51,4.09)	-1.14 (-18.96,16.68)	-0.61 (-17.82,16.59)	**Ginsenoside**			
-14.40 (-32.71,3.91)	-1.83 (-20.16,16.50)	-1.30 (-19.04,16.43)	-0.69 (-18.99,17.61)	**Lycopene**		
-15.62 (-34.46,3.22)	-3.05 (-21.90,15.80)	-2.52 (-20.80,15.75)	-1.91 (-20.73,16.91)	-1.22 (-20.53,18.09)	**Catechin**	
-17.61 (-35.38,0.16)	-5.04 (-22.83,12.75)	-4.51 (-21.69,12.66)	-3.90 (-21.66,13.86)	-3.21 (-21.48,15.06)	-1.99 (-20.79,16.81)	**TanshinoneIIA**

-17.66<br>(-34.77,-0.54)	-5.09<br>(-22.22,12.05)	-4.56<br>(-20.18,11.06)	-3.95<br>(-21.05,13.16)	-3.26<br>(-20.89,14.38)	-2.04<br>(-20.22,16.14)	-0.05<br>(-17.12,17.03)	**Luteolin**								
-17.75<br>(-32.36,-3.14)	-5.18<br>(-19.81,9.45)	-4.65<br>(-C1016.35,7.04)	-4.04<br>(-18.63,10.56)	-3.35<br>(-18.57,11.87)	-2.13<br>(-17.97,13.71)	-0.14<br>(-14.70,14.42)	-0.09<br>(-11.67,11.48)	**Sildenafil**							
-18.30<br>(-36.07,-0.53)	-5.73<br>(-23.52,12.06)	-5.20<br>(-22.38,11.98)	-4.59<br>(-22.35,13.17)	-3.90<br>(-22.18,14.38)	-2.68<br>(-21.48,16.12)	-0.69<br>(-18.42,17.04)	-0.64<br>(-17.72,16.43)	-0.55<br>(-15.11,14.01)	**APS**						
-19.22<br>(-36.36,-2.08)	-6.65<br>(-23.81,10.51)	-6.12<br>(-21.77,9.53)	-5.51<br>(-22.64,11.62)	-4.82<br>(-22.48,12.84)	-3.60<br>(-21.80,14.61)	-1.61<br>(-18.71,15.49)	-1.56<br>(-17.11,13.99)	-1.47<br>(-13.08,10.14)	-0.92<br>(-18.02,16.18)	**Paeoniflorin**					
-20.40<br>(-38.18,-2.62)	-7.83<br>(-25.63,9.97)	-7.30<br>(-24.49,9.88)	-6.69<br>(-24.46,11.08)	-6.00<br>(-24.28,12.28)	-4.78<br>(-23.58,14.02)	-2.79<br>(-20.53,14.95)	-2.74<br>(-19.83,14.34)	-2.65<br>(-17.22,11.92)	-2.10<br>(-19.84,15.64)	-1.18<br>(-18.29,15.93)	**VD3**				
-21.07<br>(-38.89,-3.25)	-8.50<br>(-21.05,4.05)	-7.97<br>(-25.21,9.26)	-7.36<br>(-25.17,10.45)	-6.67<br>(-24.99,11.65)	-5.45<br>(-24.30,13.40)	-3.46<br>(-21.24,14.32)	-3.41<br>(-20.54,13.72)	-3.32<br>(-17.95,11.31)	-2.77<br>(-20.56,15.02)	-1.85<br>(-19.01,15.30)	-0.67<br>(-18.46,17.12)	**NGFS**			
-27.10<br>(-45.00,-9.20)	-14.53<br>(-32.45,3.39)	-14.00<br>(-31.32,3.31)	-13.39<br>(-31.28,4.50)	-12.70<br>(-31.10,5.70)	-11.48<br>(-30.40,7.44)	-9.49<br>(-27.35,8.37)	-9.44<br>(-26.66,7.77)	-9.35<br>(-24.07,5.37)	-8.80<br>(-26.67,9.07)	-7.88<br>(-25.12,9.36)	-6.70<br>(-24.57,11.17)	-6.03<br>(-23.95,11.89)	**Acetazolamide**		
-27.50<br>(-45.44,-9.56)	-14.93<br>(-32.88,3.02)	-14.40<br>(-31.75,2.94)	-13.79<br>(-31.71,4.13)	-13.10<br>(-31.53,5.33)	-11.88<br>(-30.83,7.07)	-9.89<br>(-27.79,8.01)	-9.84<br>(-27.09,7.40)	-9.75<br>(-24.51,5.01)	-9.20<br>(-27.10,8.70)	-8.28<br>(-25.55,8.99)	-7.10<br>(-25.00,10.80)	-6.43<br>(-24.38,11.52)	-0.40<br>(-13.17,12.37)	**Rhodiola**	
-27.40<br>(-40.00,-14.80)	-14.83<br>(-27.45,-2.21)	-14.30<br>(-26.05,-2.56)	-13.69<br>(-26.27,-1.11)	-13.00<br>(-26.29,0.29)	-11.78<br>(-25.78,2.22)	-9.79<br>(-22.33,2.75)	-9.74<br>(-21.33,1.85)	-9.65<br>(-17.06,-2.24)	-9.10<br>(-21.64,3.44)	-8.18<br>(-19.81,3.45)	-7.00<br>(-19.55,5.55)	-6.33<br>(-18.94,6.28)	-0.30<br>(-13.02,12.42)	0.10<br>(-12.67,12.87)	**control**

Table S3.2: HIF-1

**APS**											
-2.18 (-18.73,14.36)	**ASF**										
-2.63 (-19.30,14.05)	-0.44 (-16.77,15.88)	**Polygonatum**									
-5.34 (-19.63,8.96)	-3.15 (-17.03,10.73)	-2.71 (-16.75,11.33)	**Acetazolamide**								
-6.53 (-23.01,9.96)	-4.34 (-20.47,11.78)	-3.90 (-20.16,12.36)	-1.19 (-15.00,12.62)	**Lycopene**							
-7.25 (-23.65,9.15)	-5.06 (-21.10,10.98)	-4.62 (-15.77,6.53)	-1.91 (-15.62,11.80)	-0.72 (-16.70,15.26)	**Synonyms**						
-7.80 (-24.12,8.53)	-5.61 (-16.74,5.52)	-5.17 (-21.27,10.93)	-2.46 (-16.08,11.16)	-1.27 (-17.17,14.63)	-0.55 (-16.36,15.27)	**Rutin**					
-8.02 (-22.22,6.17)	-5.84 (-19.62,7.94)	-5.40 (-19.34,8.54)	-2.69 (-13.34,7.96)	-1.50 (-15.20,12.21)	-0.78 (-14.38,12.83)	-0.23 (-13.74,13.29)	**Ginsenoside**				
-8.67 (-25.10,7.75)	-6.49 (-22.56,9.58)	-6.05 (-22.25,10.16)	-3.34 (-17.08,10.40)	-2.15 (-18.15,13.86)	-1.43 (-17.34,14.49)	-0.88 (-16.72,14.97)	-0.65 (-14.28,12.99)	**AstragalosideIV**			
-8.59 (-24.40,7.22)	-6.41 (-21.85,9.03)	-5.97 (-21.55,9.62)	-3.26 (-13.60,7.09)	-2.07 (-17.44,13.31)	-1.35 (-16.63,13.94)	-0.80 (-16.00,14.41)	-0.57 (-13.32,12.18)	0.08 (-15.23,15.39)	**L.delbrueckii**		
-8.95 (-21.90,4.01)	-6.76 (-19.26,5.73)	-6.32 (-18.99,6.35)	-3.61 (-11.92,4.70)	-2.42 (-14.84,10.00)	-1.70 (-14.00,10.60)	-1.15 (-13.36,11.05)	-0.92 (-9.04,7.20)	-0.27 (-12.61,12.07)	-0.35 (-11.47,10.76)	**Rhodiola**	
-10.31 (-25.80,5.19)	-8.12 (-23.24,6.99)	-7.68 (-22.94,7.58)	-4.97 (-17.23,7.29)	-3.78 (-18.83,11.27)	-3.06 (-18.02,11.90)	-2.51 (-17.39,12.37)	-2.28 (-14.42,9.85)	-1.63 (-16.62,13.35)	-1.71 (-15.87,12.44)	-1.36 (-11.23,8.51)	**DSP**

-11.08 (-27.44,5.28)	-8.89 (-24.89,7.11)	-8.45 (-24.59,7.69)	-5.74 (-19.40,7.92)	-4.55 (-20.49,11.39)	-3.83 (-19.68,12.02)	-3.28 (-19.06,12.49)	-3.05 (-16.61,10.51)	-2.40 (-18.28,13.48)	-2.48 (-17.73,12.76)	-2.13 (-14.38,10.12)	-0.77 (-15.69,14.15)	**Luteolin**					
-11.45 (-27.71,4.82)	-9.26 (-25.17,6.64)	-8.82 (-24.86,7.22)	-6.11 (-19.66,7.44)	-4.92 (-20.76,10.92)	-4.20 (-19.95,11.55)	-3.65 (-19.33,12.02)	-3.42 (-16.86,10.02)	-2.77 (-18.56,13.01)	-2.85 (-17.99,12.29)	-2.50 (-14.63,9.62)	-1.14 (-15.95,13.67)	-0.37 (-16.08,15.34)	**LGG**				
-12.09 (-28.37,4.19)	-9.90 (-25.82,6.02)	-9.46 (-25.52,6.60)	-6.75 (-20.32,6.82)	-5.56 (-21.41,10.30)	-4.84 (-20.61,10.93)	-4.29 (-19.98,11.40)	-4.06 (-17.52,9.40)	-3.41 (-19.21,12.38)	-3.49 (-18.65,11.66)	-3.14 (-15.28,9.00)	-1.78 (-16.61,13.05)	-1.01 (-16.74,14.72)	-0.64 (-16.27,14.99)	**Curcumin**			
-13.94 (-30.19,2.32)	-11.75 (-27.64,4.14)	-11.31 (-27.34,4.72)	-8.60 (-22.13,4.93)	-7.41 (-23.24,8.42)	-6.69 (-22.43,9.05)	-6.14 (-21.80,9.52)	-5.91 (-19.34,7.52)	-5.26 (-21.03,10.50)	-5.34 (-20.47,9.78)	-4.99 (-17.10,7.12)	-3.63 (-18.43,11.17)	-2.86 (-18.56,12.84)	-2.49 (-18.09,13.11)	-1.85 (-17.46,13.77)	**L.caseiATCC393**		
-13.41 (-25.35,-1.46)	-11.22 (-22.67,0.22)	-10.78 (-22.42,0.86)	-8.07 (-15.92,-0.22)	-6.88 (-18.24,4.48)	-6.16 (-17.40,5.08)	-5.61 (-16.74,5.52)	-5.38 (-13.05,2.29)	-4.73 (-16.01,6.54)	-4.81 (-15.17,5.55)	-4.46 (-9.47,0.55)	-3.10 (-12.97,6.77)	-2.33 (-13.51,8.85)	-1.96 (-13.00,9.08)	-1.32 (-12.38,9.74)	0.53 (-10.49,11.55)	**control**	
-16.74 (-32.42,-1.06)	-14.56 (-29.86,0.74)	-14.12 (-29.57,1.33)	-11.41 (-23.90,1.09)	-10.22 (-25.46,5.02)	-9.50 (-24.64,5.65)	-8.95 (-24.02,6.12)	-8.72 (-21.08,3.64)	-8.07 (-23.25,7.11)	-8.15 (-22.51,6.21)	-7.80 (-17.90,2.31)	-6.44 (-20.13,7.26)	-5.67 (-20.77,9.44)	-5.30 (-20.30,9.71)	-4.66 (-19.68,10.36)	-2.81 (-17.80,12.18)	-3.34 (-13.49,6.82)	**Turnip**

Table S3.3: EPO

**Nifedipine**							
-3.83 (-7.43,-0.23)	**AstragalosideIV**						
-4.12 (-7.52,-0.72)	-0.30 (-5.25,4.65)	**Arginine**					
-6.11 (-8.49,-3.72)	-2.28 (-6.60,2.04)	-1.98 (-6.14,2.17)	**Rhodiola**				
-6.74 (-8.63,-4.86)	-2.92 (-6.98,1.15)	-2.62 (-6.51,1.27)	-0.64 (-2.81,1.54)	**Ginsenoside**			
-7.10 (-9.38,-4.82)	-3.27 (-7.53,0.99)	-2.98 (-7.07,1.12)	-0.99 (-4.29,2.30)	-0.36 (-3.32,2.60)	**Acetazolamide**		
-8.83 (-11.57,-6.09)	-5.01 (-9.53,-0.48)	-4.71 (-9.07,-0.34)	-2.72 (-6.36,0.91)	-2.09 (-5.42,1.24)	-1.73 (-5.30,1.83)	**EGCG**	
-9.00 (-11.73,-6.27)	-5.18 (-9.69,-0.66)	-4.88 (-9.24,-0.52)	-2.89 (-6.52,0.73)	-2.26 (-5.58,1.06)	-1.90 (-5.46,1.66)	-0.17 (-2.50,2.16)	**control**

Table S3.4: MDA

**Ginsenoside**												
-0.05 (-4.14,4.05)	**Hyperoside**											
-1.09 (-4.94,2.77)	-1.04 (-5.68,3.59)	**MAP**										
-1.37 (-3.76,1.02)	-1.33 (-5.11,2.46)	-0.29 (-3.80,3.23)	**Rhodiola**									
-1.28 (-5.18,2.62)	-1.24 (-5.91,3.43)	-0.20 (-4.66,4.27)	0.09 (-3.48,3.66)	**Paeoniflorin**								
-1.26 (-4.07,1.55)	-1.22 (-5.70,3.26)	-0.18 (-4.44,4.09)	0.11 (-3.04,3.25)	0.02 (-4.28,4.32)	**Kaempferol**							
-1.37 (-5.34,2.59)	-1.33 (-6.06,3.40)	-0.29 (-4.81,4.23)	-0.00 (-3.64,3.64)	-0.09 (-4.65,4.47)	-0.11 (-4.47,4.25)	**Lycopene**						
-1.43 (-5.31,2.46)	-1.38 (-6.04,3.28)	-0.34 (-4.79,4.11)	-0.06 (-3.61,3.50)	-0.14 (-4.63,4.34)	-0.16 (-4.46,4.13)	-0.05 (-4.60,4.49)	**L.caseiATCC393**					
-1.72 (-5.11,1.66)	-1.68 (-6.05,2.69)	-0.64 (-4.78,3.51)	-0.35 (-3.06,2.36)	-0.44 (-4.63,3.74)	-0.46 (-4.36,3.44)	-0.35 (-4.60,3.90)	-0.30 (-4.47,3.88)	**Acteoside**				
-1.73 (-4.78,1.32)	-1.68 (-5.82,2.45)	-0.64 (-4.54,3.25)	-0.36 (-2.60,1.88)	-0.45 (-4.39,3.49)	-0.47 (-4.09,3.15)	-0.35 (-4.36,3.65)	-0.30 (-4.23,3.63)	-0.00 (-3.36,3.36)	**Turnip**			
-1.86 (-5.25,1.52)	-1.82 (-6.18,2.55)	-0.77 (-4.92,3.37)	-0.49 (-3.20,2.22)	-0.58 (-4.76,3.60)	-0.60 (-4.50,3.30)	-0.49 (-4.73,3.76)	-0.43 (-4.60,3.74)	-0.14 (-3.11,2.84)	-0.13 (-3.49,3.22)	**Echinacoside**		
-2.14 (-5.68,1.41)	-2.09 (-6.49,2.30)	-1.05 (-5.22,3.12)	-0.76 (-3.91,2.38)	-0.85 (-5.07,3.36)	-0.87 (-4.87,3.12)	-0.76 (-5.04,3.51)	-0.71 (-4.91,3.49)	-0.41 (-4.26,3.44)	-0.41 (-3.99,3.17)	-0.28 (-4.12,3.57)	**Polygonatum**	

-2.25 (-6.04,1.53)	-2.21 (-6.78,2.37)	-1.16 (-5.53,3.20)	-0.88 (-4.33,2.57)	-0.97 (-5.37,3.43)	-0.99 (-5.19,3.21)	-0.88 (-5.34,3.58)	-0.82 (-5.21,3.57)	-0.53 (-4.61,3.55)	-0.52 (-4.35,3.31)	-0.39 (-4.47,3.69)	-0.11 (-4.22,3.99)	ASF											
-2.46 (-5.93,1.00)	-2.42 (-6.75,1.92)	-1.38 (-5.48,2.73)	-1.09 (-4.15,1.97)	-1.18 (-5.33,2.97)	-1.20 (-5.13,2.73)	-1.09 (-5.30,3.12)	-1.04 (-5.17,3.10)	-0.74 (-4.52,3.04)	-0.74 (-4.24,2.77)	-0.60 (-4.38,3.18)	-0.33 (-4.03,3.37)	-0.21 (-4.26,3.83)	EGCG										
-2.46 (-5.04,0.12)	-2.42 (-6.09,1.26)	-1.37 (-4.78,2.03)	-1.09 (-3.03,0.86)	-1.18 (-4.63,2.28)	-1.20 (-4.37,1.98)	-1.09 (-4.62,2.44)	-1.03 (-4.47,2.41)	-0.74 (-3.72,2.24)	-0.73 (-3.35,1.89)	-0.60 (-3.58,2.38)	-0.32 (-3.06,2.41)	-0.21 (-3.54,3.12)	0.00 (-2.65,2.66)	Synonyms									
-2.56 (-6.23,1.11)	-2.51 (-7.01,1.98)	-1.47 (-5.75,2.81)	-1.19 (-4.49,2.11)	-1.28 (-5.60,3.04)	-1.30 (-5.40,2.81)	-1.18 (-5.56,3.19)	-1.13 (-5.44,3.17)	-0.83 (-4.81,3.14)	-0.83 (-4.53,2.86)	-0.70 (-4.67,3.27)	-0.42 (-4.44,3.59)	-0.31 (-4.52,3.91)	-0.09 (-4.05,3.86)	-0.10 (-3.31,3.11)	L.delbrueckii								
-2.89 (-6.65,0.87)	-2.85 (-7.40,1.71)	-1.80 (-6.14,2.54)	-1.52 (-4.94,1.90)	-1.61 (-5.99,2.77)	-1.63 (-5.81,2.55)	-1.52 (-5.96,2.92)	-1.46 (-5.83,2.91)	-1.17 (-5.22,2.89)	-1.16 (-4.96,2.64)	-1.03 (-5.08,3.03)	-0.75 (-4.84,3.33)	-0.64 (-3.62,2.34)	-0.43 (-4.45,3.59)	-0.43 (-3.73,2.87)	-0.33 (-4.53,3.86)	Rutin							
-2.87 (-5.75,0.01)	-2.83 (-6.70,1.04)	-1.78 (-5.40,1.83)	-1.50 (-3.89,0.89)	-1.59 (-5.25,2.07)	-1.61 (-5.02,1.80)	-1.50 (-5.23,2.24)	-1.44 (-5.09,2.20)	-1.15 (-4.40,2.11)	-1.14 (-4.06,1.77)	-1.01 (-4.26,2.24)	-0.73 (-4.03,2.57)	-0.62 (-4.16,2.92)	-0.41 (-3.63,2.81)	-0.41 (-2.67,1.85)	-0.31 (-3.16,2.53)	0.02 (-3.49,3.53)	Acetazolamide						
-2.99 (-6.55,0.58)	-2.94 (-7.34,1.46)	-1.90 (-6.08,2.28)	-1.61 (-4.81,1.58)	-1.70 (-5.92,2.52)	-1.72 (-5.73,2.28)	-1.61 (-5.89,2.67)	-1.56 (-5.77,2.65)	-1.26 (-5.14,2.62)	-1.26 (-4.86,2.35)	-1.12 (-5.00,2.75)	-0.85 (-4.76,3.06)	-0.73 (-4.85,3.38)	-0.52 (-4.36,3.32)	-0.53 (-3.61,2.55)	-0.43 (-4.21,3.36)	-0.10 (-4.19,4.00)	-0.12 (-2.88,2.65)	Crocetin					
-3.17 (-6.93,0.59)	-3.13 (-7.68,1.43)	-2.08 (-6.43,2.26)	-1.80 (-5.22,1.62)	-1.89 (-6.27,2.49)	-1.91 (-6.09,2.27)	-1.80 (-6.24,2.64)	-1.74 (-6.11,2.63)	-1.45 (-5.51,2.61)	-1.44 (-5.25,2.36)	-1.31 (-5.37,2.75)	-1.03 (-5.12,3.05)	-0.92 (-5.20,3.36)	-0.71 (-4.73,3.31)	-0.71 (-4.01,2.59)	-0.61 (-4.81,3.58)	-0.28 (-4.54,3.98)	-0.30 (-3.82,3.22)	-0.18 (-4.28,3.91)	LGG				
-3.40 (-7.14,0.35)	-3.35 (-7.90,1.19)	-2.31 (-5.36,0.73)	-2.03 (-5.43,1.37)	-2.12 (-6.49,2.25)	-2.14 (-6.30,2.03)	-2.03 (-6.45,2.40)	-1.97 (-6.33,2.38)	-1.68 (-5.72,2.37)	-1.67 (-5.46,2.12)	-1.54 (-5.58,2.50)	-1.26 (-5.33,2.81)	-1.15 (-5.42,3.12)	-0.94 (-4.94,3.07)	-0.94 (-4.22,2.34)	-0.84 (-5.02,3.34)	-0.51 (-4.75,3.74)	-0.53 (-4.03,2.97)	-0.41 (-4.49,3.67)	-0.23 (-4.48,4.02)	Atorvastatin			
-3.58 (-7.14,-0.03)	-3.54 (-7.94,0.86)	-2.50 (-6.67,1.68)	-2.21 (-5.37,0.94)	-2.30 (-6.52,1.92)	-2.32 (-6.32,1.68)	-2.21 (-6.49,2.07)	-2.16 (-6.36,2.05)	-1.86 (-5.72,2.00)	-1.86 (-5.45,1.73)	-1.72 (-5.58,2.13)	-1.45 (-5.23,2.34)	-1.33 (-5.45,2.78)	-1.12 (-4.84,2.60)	-1.12 (-3.89,1.65)	-1.03 (-5.05,3.00)	-0.69 (-4.78,3.40)	-0.71 (-4.02,2.59)	-0.60 (-4.52,3.32)	-0.41 (-4.51,3.68)	-0.19 (-4.26,3.89)	DSP		
-3.99 (-7.55,-0.44)	-3.95 (-8.34,0.45)	-2.90 (-7.07,1.26)	-2.62 (-5.80,0.56)	-2.71 (-6.92,1.50)	-2.73 (-6.72,1.27)	-2.62 (-6.89,1.65)	-2.56 (-6.76,1.63)	-2.27 (-6.13,1.60)	-2.26 (-5.85,1.33)	-2.13 (-5.99,1.73)	-1.85 (-5.75,2.05)	-1.74 (-5.84,2.37)	-1.53 (-5.36,2.31)	-1.53 (-4.60,1.54)	-1.43 (-5.21,2.35)	-1.10 (-5.18,2.98)	-1.12 (-3.88,1.64)	-1.00 (-4.70,2.69)	-0.82 (-4.90,3.26)	-0.59 (-4.66,3.48)	-0.41 (-4.31,3.50)	Puerarin	
-4.57 (-6.83,-2.32)	-4.53 (-7.95,-1.11)	-3.49 (-6.61,-0.36)	-3.20 (-4.82,-1.59)	-3.29 (-6.47,-0.11)	-3.31 (-6.21,-0.41)	-3.20 (-6.46,0.06)	-3.15 (-6.31,0.02)	-2.85 (-5.57,-0.13)	-2.85 (-5.17,-0.52)	-2.71 (-5.43,0.00)	-2.44 (-5.20,0.32)	-2.32 (-5.37,0.72)	-2.11 (-4.77,0.55)	-2.11 (-3.46,-0.77)	-2.02 (-4.94,0.90)	-1.68 (-4.70,1.33)	-1.70 (-3.52,0.11)	-1.59 (-4.36,1.18)	-1.40 (-4.42,1.61)	-1.18 (-4.17,1.82)	-0.99 (-3.76,1.78)	-0.58 (-3.34,2.17)	control

Table S3.5: SOD

**GP**												
0.30 (-5.55,6.14)	**Ginsenoside**											
0.87 (-3.32,5.06)	0.57 (-3.66,4.80)	**Rhodiola**										
0.61 (-6.09,7.30)	0.31 (-6.41,7.03)	-0.26 (-5.75,5.23)	**UrsolicAcid**									
1.05 (-5.31,7.41)	0.75 (-5.63,7.13)	0.18 (-4.89,5.25)	0.44 (-6.57,7.45)	**Atorvastatin**								
1.49 (-3.17,6.15)	1.19 (-3.50,5.89)	0.62 (-1.96,3.21)	0.89 (-4.68,6.45)	0.44 (-4.71,5.60)	**Synonyms**							
1.64 (-3.54,6.81)	1.34 (-3.87,6.55)	0.77 (-2.58,4.12)	1.03 (-5.04,7.10)	0.59 (-5.10,6.28)	0.15 (-3.59,3.88)	**Turnip**						
1.84 (-3.92,7.59)	1.54 (-4.24,7.32)	0.97 (-3.15,5.09)	1.23 (-5.39,7.85)	0.79 (-5.49,7.06)	0.34 (-4.21,4.90)	0.20 (-4.89,5.29)	**Acteoside**					
1.95 (-3.80,7.70)	1.65 (-4.13,7.43)	1.08 (-3.04,5.20)	1.34 (-5.27,7.96)	0.90 (-5.37,7.17)	0.46 (-4.10,5.01)	0.31 (-4.78,5.40)	0.11 (-4.52,4.75)	**Echinacoside**				
2.22 (-4.13,8.56)	1.92 (-4.45,8.29)	1.35 (-3.71,6.41)	1.61 (-5.39,8.61)	1.17 (-5.51,7.85)	0.72 (-4.42,5.87)	0.58 (-5.10,6.26)	0.38 (-5.88,6.65)	0.27 (-6.00,6.53)	**Hyperoside**			
2.17 (-3.77,8.11)	1.87 (-4.09,7.84)	1.30 (-3.22,5.82)	1.57 (-5.09,8.22)	1.12 (-5.19,7.44)	0.68 (-3.49,4.85)	0.53 (-4.70,5.77)	0.34 (-5.52,6.19)	0.22 (-5.63,6.08)	-0.04 (-6.35,6.26)	**Polygonatum**		
2.52 (-3.59,8.62)	2.22 (-3.91,8.35)	1.65 (-3.10,6.40)	1.91 (-4.87,8.70)	1.47 (-4.98,7.92)	1.03 (-3.81,5.87)	0.88 (-4.53,6.29)	0.68 (-5.34,6.70)	0.57 (-5.45,6.59)	0.30 (-6.14,6.74)	0.35 (-5.72,6.41)	**Crocetin**	

2.48 (-2.81,7.77)	2.18 (-3.13,7.50)	1.61 (-2.02,5.24)	1.87 (-4.20,7.94)	1.43 (-4.27,7.12)	0.99 (-2.45,4.42)	0.84 (-3.63,5.31)	0.64 (-4.55,5.83)	0.53 (-4.66,5.72)	0.26 (-5.42,5.95)	0.31 (-4.83,5.44)	-0.04 (-5.45,5.38)	**DSP**										
2.58 (-3.76,8.93)	2.29 (-4.08,8.65)	1.71 (-3.34,6.77)	1.98 (-5.03,8.98)	1.53 (-5.14,8.21)	1.09 (-4.05,6.23)	0.95 (-4.73,6.63)	0.75 (-5.52,7.01)	0.63 (-5.63,6.90)	0.37 (-6.30,7.04)	0.41 (-5.89,6.72)	0.07 (-6.38,6.51)	0.11 (-5.58,5.79)	**Kaempferol**									
2.62 (-3.70,8.94)	2.32 (-4.02,8.66)	1.75 (-3.27,6.78)	2.01 (-4.96,8.99)	1.57 (-5.08,8.22)	1.13 (-3.98,6.23)	0.98 (-4.67,6.63)	0.78 (-5.45,7.02)	0.67 (-5.56,6.91)	0.40 (-6.24,7.05)	0.45 (-5.83,6.73)	0.10 (-6.31,6.52)	0.14 (-5.51,5.79)	0.04 (-6.60,6.68)	**LGG**								
2.95 (-3.36,9.26)	2.65 (-3.68,8.98)	2.08 (-2.93,7.09)	2.34 (-4.63,9.31)	1.90 (-4.74,8.54)	1.46 (-3.64,6.55)	1.31 (-4.33,6.95)	1.11 (-5.11,7.34)	1.00 (-5.23,7.22)	0.73 (-5.90,7.37)	0.78 (-5.49,7.04)	0.43 (-5.97,6.83)	0.47 (-5.17,6.11)	0.36 (-6.27,7.00)	0.33 (-6.28,6.93)	**Paeoniflorin**							
3.19 (-3.11,9.49)	2.89 (-3.43,9.22)	2.32 (-2.68,7.32)	2.58 (-4.38,9.54)	2.14 (-4.50,8.78)	1.70 (-3.39,6.78)	1.55 (-4.08,7.18)	1.35 (-4.87,7.57)	1.24 (-4.98,7.46)	0.97 (-5.66,7.60)	1.02 (-5.25,7.28)	0.67 (-5.73,7.07)	0.71 (-4.92,6.34)	0.60 (-6.02,7.23)	0.57 (-6.03,7.17)	0.24 (-6.35,6.83)	**L.caseiATCC393**						
3.08 (-2.32,8.49)	2.79 (-2.65,8.22)	2.22 (-1.60,6.03)	2.48 (-3.69,8.64)	2.03 (-3.76,7.83)	1.59 (-2.33,5.52)	1.45 (-3.16,6.06)	1.25 (-4.06,6.56)	1.13 (-4.18,6.45)	0.87 (-4.92,6.65)	0.91 (-4.45,6.28)	0.57 (-3.81,4.95)	0.61 (-4.01,5.22)	0.50 (-5.28,6.29)	0.46 (-5.29,6.22)	0.14 (-5.61,5.88)	-0.10 (-5.84,5.63)	**Acetazolamide**					
3.54 (-2.75,9.83)	3.24 (-3.07,9.56)	2.67 (-2.32,7.66)	2.93 (-4.02,9.88)	2.49 (-2.20,7.17)	2.05 (-3.03,7.12)	1.90 (-3.72,7.52)	1.70 (-4.51,7.91)	1.59 (-4.62,7.80)	1.32 (-5.30,7.94)	1.37 (-4.89,7.62)	1.02 (-5.37,7.41)	1.06 (-4.56,6.68)	0.96 (-5.66,7.57)	0.92 (-5.67,7.51)	0.59 (-5.99,7.17)	0.35 (-6.22,6.93)	0.45 (-5.27,6.18)	**MAP**				
3.78 (-2.35,9.90)	3.48 (-2.67,9.63)	2.91 (-1.87,7.69)	3.17 (-3.63,9.97)	2.73 (-3.74,9.20)	2.29 (-2.58,7.15)	2.14 (-3.29,7.57)	1.94 (-4.10,7.98)	1.83 (-4.21,7.87)	1.56 (-4.90,8.02)	1.61 (-4.48,7.69)	1.26 (-4.49,7.01)	1.30 (-4.14,6.74)	1.19 (-5.27,7.65)	1.16 (-5.28,7.59)	0.83 (-5.59,7.25)	0.59 (-5.83,7.01)	0.69 (-3.72,5.11)	0.24 (-6.17,6.64)	**L.delbrueckii**			
4.13 (-1.64,9.89)	3.83 (-1.96,9.62)	3.26 (-0.88,7.40)	3.52 (-3.11,10.15)	3.08 (-3.21,9.36)	2.64 (-1.93,7.21)	2.49 (-2.61,7.60)	2.29 (-3.40,7.99)	2.18 (-3.52,7.87)	1.91 (-4.36,8.19)	1.96 (-3.91,7.83)	1.61 (-4.42,7.64)	1.65 (-3.55,6.85)	1.54 (-4.73,7.82)	1.51 (-4.74,7.75)	1.18 (-5.06,7.42)	0.94 (-5.29,7.17)	1.04 (-4.28,6.37)	0.59 (-5.63,6.81)	0.35 (-5.70,6.40)	**CRB**		
4.46 (0.21,8.71)	4.16 (-0.12,8.44)	3.59 (1.76,5.42)	3.85 (-1.32,9.03)	3.41 (-1.32,8.14)	2.97 (0.92,5.01)	2.82 (-0.34,5.99)	2.62 (-1.50,6.75)	2.51 (-1.61,6.63)	2.24 (-2.47,6.96)	2.29 (-1.90,6.48)	1.94 (-2.45,6.33)	1.98 (-1.19,5.16)	1.88 (-2.84,6.59)	1.84 (-2.84,6.52)	1.51 (-3.15,6.18)	1.27 (-3.38,5.93)	1.38 (-1.97,4.73)	0.92 (-3.72,5.56)	0.68 (-3.73,5.10)	0.33 (-3.81,4.47)	**control**	
7.31 (0.95,13.67)	7.02 (0.63,13.40)	6.44 (1.36,11.52)	6.71 (-0.31,13.72)	6.26 (-0.43,12.96)	5.82 (0.66,10.98)	5.68 (-0.02,11.37)	5.48 (-0.80,11.76)	5.36 (-0.92,11.64)	5.10 (-1.59,11.78)	5.14 (-1.18,11.46)	4.79 (-1.66,11.25)	4.83 (-0.87,10.54)	4.73 (-1.95,11.41)	4.69 (-1.97,11.35)	4.36 (-2.28,11.01)	4.12 (-2.52,10.77)	4.23 (-1.57,10.03)	3.77 (-2.86,10.41)	3.54 (-2.94,10.01)	3.18 (-3.11,9.48)	2.85 (-1.89,7.59)	**Cartialgenous**

Table S3.6: GSH

**Rhodiola**												
0.02 (-4.96,5.01)	**Ginsenoside**											
-0.15 (-8.06,7.77)	-0.17 (-8.91,8.58)	**UrsolicAcid**										
1.13 (-4.91,7.17)	1.11 (-6.39,8.61)	1.28 (-8.29,10.85)	**Turnip**									
1.21 (-4.46,6.87)	1.19 (-5.59,7.97)	1.35 (-7.51,10.21)	0.08 (-7.74,7.89)	**Acetazolamide**								
1.36 (-3.54,6.27)	1.34 (-5.07,7.76)	1.51 (-7.16,10.18)	0.23 (-7.20,7.66)	0.16 (-6.53,6.84)	**Synonyms**							
1.51 (-4.50,7.53)	1.49 (-5.96,8.95)	1.66 (-7.86,11.18)	0.38 (-7.92,8.68)	0.31 (-7.45,8.06)	0.15 (-7.23,7.53)	**CRB**						
1.77 (-5.23,8.77)	1.75 (-6.18,9.68)	1.92 (-7.85,11.69)	0.64 (-8.19,9.47)	0.57 (-5.76,6.89)	0.41 (-7.44,8.26)	0.26 (-8.52,9.04)	**Puerarin**					
1.86 (-4.16,7.87)	1.84 (-5.61,9.29)	2.00 (-7.52,11.53)	0.73 (-7.57,9.02)	0.65 (-7.11,8.40)	0.49 (-6.89,7.87)	0.34 (-7.92,8.61)	0.08 (-8.69,8.86)	**Acteoside**				
2.17 (-3.84,8.19)	2.15 (-5.30,9.60)	2.32 (-7.20,11.84)	1.04 (-7.26,9.34)	0.97 (-6.79,8.72)	0.81 (-6.57,8.19)	0.66 (-7.60,8.92)	0.40 (-8.38,9.18)	0.32 (-6.43,7.06)	**Echinacoside**			
2.26 (-4.70,9.23)	2.24 (-5.65,10.14)	2.41 (-7.33,12.15)	1.13 (-7.67,9.93)	1.05 (-5.29,7.40)	0.90 (-6.92,8.71)	0.75 (-8.00,9.50)	0.49 (-7.80,8.78)	0.40 (-8.34,9.15)	0.09 (-8.66,8.83)	**Crocetin**		
5.05 (2.18,7.92)	5.03 (0.34,9.72)	5.20 (-2.18,12.58)	3.92 (-2.17,10.01)	3.84 (-1.06,8.74)	3.69 (-0.87,8.24)	3.54 (-2.48,9.56)	3.28 (-3.12,9.67)	3.19 (-2.82,9.21)	2.88 (-3.14,8.89)	2.79 (-3.56,9.14)	**control**	

Table S3.7: LA

**Moringa**									
-0.82(-3.63,1.98)	**Resveratrol**								
-1.48(-3.68,0.72)	-0.66(-3.04,1.73)	**Acteoside**							
-1.73(-3.92,0.46)	-0.90(-3.28,1.47)	-0.25(-1.85,1.35)	**Curcumin**						
-1.86(-4.05,0.33)	-1.03(-3.41,1.34)	-0.38(-1.87,1.12)	-0.13(-1.71,1.45)	**CRB**					
-2.11(-4.11,-0.10)	-1.28(-3.49,0.92)	-0.63(-1.73,0.48)	-0.38(-1.68,0.92)	-0.25(-1.34,0.84)	**Rhodiola**				
-2.29(-4.59,0.02)	-1.46(-3.95,1.03)	-0.81(-2.56,0.95)	-0.56(-2.25,1.14)	-0.43(-2.17,1.32)	-0.18(-1.67,1.31)	**DSP**			
-2.27(-4.44,-0.09)	-1.44(-3.80,0.92)	-0.78(-1.98,0.41)	-0.54(-2.10,1.02)	-0.41(-1.88,1.06)	-0.16(-1.24,0.92)	0.02(-1.70,1.75)	**Echinacoside**		
-2.50(-4.48,-0.52)	-1.67(-3.86,0.51)	-1.02(-2.30,0.26)	-0.77(-1.89,0.34)	-0.64(-1.90,0.62)	-0.39(-1.25,0.47)	-0.21(-1.56,1.13)	-0.23(-1.47,1.00)	**Synonyms**	
-3.51(-5.39,-1.64)	-2.69(-4.78,-0.60)	-2.03(-3.18,-0.89)	-1.79(-2.92,-0.65)	-1.66(-2.78,-0.53)	-1.41(-2.11,-0.70)	-1.23(-2.58,0.12)	-1.25(-2.35,-0.15)	-1.02(-1.66,-0.37)	**control**

Table S3.8: Glycogen

**Moringa**						
0.36 (0.20,0.52)	**Acteoside**					
0.34 (0.10,0.57)	-0.03 (-0.22,0.17)	**DSP**				
0.42 (0.26,0.57)	0.05 (-0.02,0.12)	0.08 (-0.11,0.27)	**Rhodiola**			
0.47 (0.33,0.62)	0.11 (0.04,0.18)	0.14 (-0.05,0.32)	0.06 (0.01,0.11)	**Synonyms**		
0.47 (0.31,0.64)	0.11 (0.03,0.19)	0.14 (-0.06,0.34)	0.06 (-0.02,0.13)	-0.00 (-0.08,0.08)	**Echinacoside**	
0.51 (0.36,0.66)	0.15 (0.08,0.21)	0.17 (-0.01,0.36)	0.09 (0.05,0.14)	0.04 (0.00,0.07)	0.04 (-0.03,0.11)	**control**

Table S3.9: LDH

**VD3**													
-3.33 (-7.93,1.28)	**Rhodiola**												
-3.96 (-9.04,1.11)	-0.64 (-4.68,3.40)	**MAP**											
-4.06 (-9.13,1.01)	-0.73 (-4.77,3.31)	-0.09 (-4.66,4.47)	**Rutin**										
-4.32 (-9.24,0.60)	-1.00 (-3.97,1.98)	-0.36 (-4.76,4.04)	-0.26 (-4.66,4.13)	**Acteoside**									
-4.44 (-9.31,0.42)	-1.12 (-4.89,2.65)	-0.48 (-4.81,3.85)	-0.39 (-4.71,3.94)	-0.12 (-4.27,4.03)	**EGCG**								
-4.65 (-8.99,-0.30)	-1.32 (-4.39,1.75)	-0.68 (-4.43,3.06)	-0.59 (-4.33,3.15)	-0.33 (-3.86,3.20)	-0.20 (-3.07,2.66)	**Synonyms**							
-4.82 (-9.73,0.10)	-1.49 (-4.46,1.48)	-0.85 (-5.25,3.54)	-0.76 (-5.15,3.63)	-0.50 (-3.63,2.64)	-0.37 (-4.52,3.77)	-0.17 (-3.69,3.35)	**Echinacoside**						
-5.01 (-10.07,0.06)	-1.68 (-5.71,2.35)	-1.04 (-5.60,3.52)	-0.95 (-5.50,3.61)	-0.68 (-5.07,3.70)	-0.56 (-4.88,3.76)	-0.36 (-4.09,3.38)	-0.19 (-4.57,4.20)	**Hyperoside**					
-5.30 (-10.19,-0.41)	-1.97 (-5.77,1.83)	-1.33 (-5.70,3.03)	-1.24 (-5.60,3.12)	-0.98 (-5.16,3.20)	-0.85 (-4.73,3.02)	-0.65 (-3.56,2.26)	-0.48 (-4.65,3.69)	-0.29 (-4.64,4.06)	**Polygonatum**				
-5.75 (-10.77,-0.73)	-2.43 (-6.40,1.55)	-1.79 (-4.96,1.38)	-1.70 (-6.20,2.81)	-1.43 (-5.77,2.91)	-1.31 (-5.58,2.96)	-1.10 (-4.78,2.57)	-0.93 (-5.27,3.40)	-0.75 (-5.25,3.75)	-0.45 (-4.76,3.85)	**Atorvastatin**			
-5.77 (-10.68,-0.85)	-2.44 (-6.27,1.39)	-1.80 (-6.19,2.59)	-1.71 (-6.10,2.68)	-1.44 (-5.66,2.77)	-1.32 (-5.24,2.59)	-1.12 (-4.09,1.86)	-0.95 (-5.15,3.26)	-0.76 (-5.14,3.62)	-0.47 (-4.42,3.48)	-0.01 (-4.34,4.32)	**DSP**		
-6.29 (-11.30,-1.27)	-2.96 (-6.93,1.01)	-2.32 (-6.83,2.18)	-2.23 (-5.42,0.96)	-1.97 (-6.30,2.37)	-1.84 (-6.11,2.42)	-1.64 (-5.30,2.03)	-1.47 (-5.79,2.86)	-1.28 (-5.78,3.21)	-0.99 (-5.28,3.31)	-0.53 (-4.98,3.91)	-0.52 (-4.84,3.80)	**ASF**	
-6.81 (-10.72,-2.90)	-3.49 (-5.91,-1.06)	-2.85 (-6.08,0.38)	-2.76 (-5.98,0.47)	-2.49 (-5.48,0.49)	-2.37 (-5.25,0.52)	-2.16 (-4.06,-0.27)	-1.99 (-4.97,0.98)	-1.81 (-5.02,1.41)	-1.51 (-4.45,1.42)	-1.06 (-4.21,2.09)	-1.05 (-4.02,1.93)	-0.53 (-3.66,2.61)	**control**

Sections S6: SYRCLE risk of bias assessment results

		selection bias		performance bias	attrition bias	reporting bias	other bias
Author	Year	Random sequence generation	baseline characteristics	masking	Incomplete outcome data	Selective reporting	other bias
Anjiati	2020	3	1	1	1	1	1
Liu	2020	2	1	1	3	1	1
Qin	2022	2	1	1	2	1	1
Ren	2022	2	1	1	1	1	1
Wang	2024	1	1	1	3	1	1
Wang	2021	3	1	1	2	1	1
Wangzixi	2024	2	1	1	2	1	1
Yu	2022	2	1	1	2	1	1
Tang	2023	2	1	1	2	1	1
Bao	2020	2	1	1	2	1	1
Cao	2022	2	1	1	2	1	1
Chen	2022	2	1	1	1	1	1
Chen	2023	2	1	1	1	1	1
Wang	2023	2	1	1	2	1	1
Wang	2022	2	1	1	2	2	1
Chu	2023	2	1	1	1	1	1
Dai	2024	2	1	1	1	1	1
Deng	2022	2	1	1	2	1	1
Dou	2023	2	1	1	2	1	1

		selection bias		performance bias	attrition bias	reporting bias	other bias
Author	Year	**Random sequence generation**	**baseline characteristics**	**masking**	**Incomplete outcome data**	**Selective reporting**	**other bias**
Guo	2023	2	1	1	1	1	1
He	2019	2	1	1	2	1	1
He	2021	1	1	1	1	1	1
Hu	2021	2	1	1	2	1	1
Huo	2021	2	1	1	2	1	1
Arti	2023	2	1	1	1	1	1
Ji	2022	2	1	1	2	1	1
Ji	2024	2	1	1	1	1	1
Song	2024	2	1	1	2	1	1
Li	2022	2	1	1	2	1	1
Li	2023	2	2	1	1	1	1
Liu	2025	1	1	1	1	1	1
Liu	2022	2	1	1	2	1	1
Long	2021	3	2	1	2	1	1
Titto	2020	2	1	1	1	1	1
Ma	2022	2	1	1	2	1	1
Ma	2020	2	1	1	1	1	1
Ni	2019	2	1	1	1	1	1
QinN	2022	2	1	1	2	1	1
Richa	2021	2	1	1	1	1	1
Ren	2024	2	1	1	2	1	1

		selection bias		performance bias	attrition bias	reporting bias	other bias
Author	Year	**Random sequence generation**	**baseline characteristics**	**masking**	**Incomplete outcome data**	**Selective reporting**	**other bias**
Wang	2020	2	1	1	2	1	1
WangZ	2022	1	1	1	1	1	1
LiX	2023	2	1	1	1	1	1
Yan	2019	2	1	1	1	1	1
Yang	2024	2	1	1	2	1	1
YangX	2024	1	1	1	1	1	1
Yu	2023	2	1	1	2	1	1
zhang	2023	2	1	1	1	1	1
Zou	2022	2	1	1	1	1	1
